# Systematic comparative analysis of single-nucleotide variant detection methods from single-cell RNA sequencing data

**DOI:** 10.1186/s13059-019-1863-4

**Published:** 2019-11-19

**Authors:** Fenglin Liu, Yuanyuan Zhang, Lei Zhang, Ziyi Li, Qiao Fang, Ranran Gao, Zemin Zhang

**Affiliations:** 1 School of Life Sciences and BIOPIC, Peking University, Beijing, China; 2 Beijing Advanced Innovation Centre for Genomics, Peking-Tsinghua Centre for Life Sciences, Peking University, Beijing, China

**Keywords:** Single-nucleotide variant detection, Somatic mutations, Single-cell RNA sequencing, Benchmarking

## Abstract

**Background:**

Systematic interrogation of single-nucleotide variants (SNVs) is one of the most promising approaches to delineate the cellular heterogeneity and phylogenetic relationships at the single-cell level. While SNV detection from abundant single-cell RNA sequencing (scRNA-seq) data is applicable and cost-effective in identifying expressed variants, inferring sub-clones, and deciphering genotype-phenotype linkages, there is a lack of computational methods specifically developed for SNV calling in scRNA-seq. Although variant callers for bulk RNA-seq have been sporadically used in scRNA-seq, the performances of different tools have not been assessed.

**Results:**

Here, we perform a systematic comparison of seven tools including SAMtools, the GATK pipeline, CTAT, FreeBayes, MuTect2, Strelka2, and VarScan2, using both simulation and scRNA-seq datasets, and identify multiple elements influencing their performance. While the specificities are generally high, with sensitivities exceeding 90% for most tools when calling homozygous SNVs in high-confident coding regions with sufficient read depths, such sensitivities dramatically decrease when calling SNVs with low read depths, low variant allele frequencies, or in specific genomic contexts. SAMtools shows the highest sensitivity in most cases especially with low supporting reads, despite the relatively low specificity in introns or high-identity regions. Strelka2 shows consistently good performance when sufficient supporting reads are provided, while FreeBayes shows good performance in the cases of high variant allele frequencies.

**Conclusions:**

We recommend SAMtools, Strelka2, FreeBayes, or CTAT, depending on the specific conditions of usage. Our study provides the first benchmarking to evaluate the performances of different SNV detection tools for scRNA-seq data.

## Background

Substantial genetic variations accumulate during tumorigenesis, leading to genetically divergent subpopulations. SNVs could be faithfully propagated from progenitors to daughter cells during DNA replication and thus have been commonly used to delineate the heterogeneity and phylogenetic relationship of tumor cells [1–4]. Next generation sequencing is by far the most useful technology to detect mutations for its ability to screen SNVs in a high-throughput manner. SNVs could be detected from the whole genome sequencing (WGS) or whole exome sequencing (WES), and then be utilized to infer clonal architecture or to construct the evolutionary relationships of tumors [5].

Accordingly, in single-cell studies, SNV detection is a compelling strategy to decipher the heterogeneity of cell compositions and to infer the lineage relationships. Although single-cell WGS (scWGS) or single-cell WES (scWES) experiments could be performed to detect single-cell SNVs [6–8], there are substantial challenges which hamper the large-scale application of such experiments. Indeed, the sparse distribution of SNVs throughout the genome might lead to a substantial proportion of SNVs undetected in single-cell experiments. In addition, numerous biases can be introduced by amplification methods that could be error prone, thus failing to provide equal coverage across the genome. Furthermore, the relatively high cost of these experiments hinders the large-scale application of such methods [9].

In contrast, scRNA-seq has been recently exploding with the continuous technological innovation and constantly increasing throughput with decreasing costs. It has been widely used for its capability of revealing complex and rare cell populations, uncovering regulatory relationships between genes, and tracking the trajectories of distinct cell lineages in development [10, 11]. Most of these analyses were based on the transcriptome data with quantified gene expression as features, which could be influenced by different technical factors such as sequencing platforms, batch effects, and dropouts, while the detection of genetic variations such as SNVs is orthogonal to such expression-based analysis, thus potentially increasing the value of the rich resource of scRNA-seq data. Importantly, SNVs may help to unravel the heterogeneity of tumors [12] and genotype-phenotype associations [13]. When considering diseases including cancer, expressed mutations are of greater interest because they could affect cellular processes more directly and their functions are more clearly illustrated. In addition, detecting SNVs from scRNA-seq data with quantified expression further enables the study of allelic expression [14] and manifests the effects of SNVs on gene expression by cis and/or trans effect [15, 16]. Furthermore, identifying SNVs from scRNA-seq could be used to find RNA-editing events and to validate DNA sequence variations. Recently, the mitochondria DNA mutations derived from scRNA-seq were reported to be a powerful and scalable strategy to assess cellular dynamics of native human cells, thus providing a natural barcode to infer clonal relationships [17]. Therefore, detecting variants from scRNA-seq data seems to be a powerful and cost-effective approach, which could not only identify the expressed variants directly, but also simultaneously reveal the relationships of DNA alteration and RNA expression at the single-cell level.

In spite of its importance, significant challenges exist for detecting variants from scRNA-seq data. The sequencing coverages are usually limited, and it is more difficult to detect variants from the transcriptome than from DNA sequences due to RNA splicing. In addition, the dynamic nature of RNAs, the higher error rate from reverse transcription, and the greater number of PCR cycles could lead to false positives. Despite these challenges, SNV detection based on scRNA-seq data has been performed by existing methods that were originally developed for bulk RNA-seq data [13, 18–20], due to the lack of tools specifically designated for scRNA-seq. However, the accuracies and specificities of these methods need to be evaluated considering the enormous challenges of RNA-based SNV detection. Indeed, while benchmarking for detecting SNVs from bulk RNA-seq data has been performed with limited tools and methods [21], there is no systematic comparison of SNV detection from scRNA-seq to our knowledge. Therefore, it is of paramount importance to evaluate the performance of variant detection tools employed in RNA-seq data at single-cell level.

In this study, we perform systematic comparative analysis of seven widely used SNV-calling methods, including SAMtools, the GATK Best Practices pipeline, CTAT, FreeBayes, MuTect2, Strelka2, and VarScan2, on both simulated and real single-cell RNA-seq datasets. We evaluate the performances of these tools in different read depths, genomic contexts, functional regions, and variant allele frequencies. We also investigate the consistency of performances for different tissue origins, as well as the impact of sequencing protocols. This study can serve as a valuable benchmark and guideline for selecting the suitable software for SNV detection in scRNA-seq.

## Results

### Overview of variant-calling methods for RNA sequencing data

The conventional SNV-calling pipeline for high-throughput transcriptome sequencing generally consists of four components: mapping, pre-processing, variant evaluation, and post-filtering. RNA-seq reads are usually mapped to the reference genome using a splice-aware mapper. The Spliced Transcripts Alignment to a Reference (STAR) aligner is recommended by the Genome Analysis Toolkit (GATK) Best Practices [22]. It performs sequential maximum mappable seed searches, seed clustering, and stitching [23]. Genomic Short-read Nucleotide Alignment Program (GSNAP) is also widely used in scRNA-seq for its tolerance of complex variants and splicing for both short and long sequence reads [24]. Pre-processing procedures, including removing duplicates, realigning, reassigning mapping qualities, and recalibrating base quality scores, could eliminate low-quality reads and improve the accuracy of variant calling. Variant evaluation is the key step, in which reliable candidates are obtained with the best performing software for downstream analysis, and thus, it is the focus of our benchmarking. Post-filtering aims to rule out the false-positive calls from diverse sources, including low quality (probability) of SNVs, low-complexity regions, and low read depths, and to retain high-confident SNVs.

MuTect2, Strelka2, and VarScan2 have been widely used to detect variants in bulk RNA-seq data. MuTect2 combines the DREAM challenge-winning somatic genotyping engine with HaplotypeCaller, allowing for a varying allelic fraction and several harder filters [25, 26]. Strelka2 utilizes mixture model-based parameter estimation and an efficient tiered haplotype-modeling strategy for variant detection [27]. VarScan2 applies a heuristic and statistical algorithm to detect and classify sequence variants [28]. Although these three tools have not been used for single-cell SNV detection, we included them in our benchmarking of scRNA-seq, considering their extensive utilization.

The GATK Best Practices for variant calling on RNA-seq data is the most frequently used framework for detecting variations in single-cell RNA-seq, in which there are two tools for variant evaluation, UnifiedGenotyper and HaplotypeCaller [18, 19, 29, 30]. HaplotypeCaller is more recent and sophisticated and is recommended by GATK. Notably, Trinity Cancer Transcriptome Analysis Toolkit (CTAT), the software developed for scRNA-seq SNV detection, was based on the GATK Best Practices pipeline. In addition, SSrGE, developed to link effective and expressed nucleotide variations associated with gene expression in scRNA-seq data, utilizes a module for identifying variants based on GATK [13].

Apart from the GATK framework, SAMtools has also been used to examine SNVs in scRNA-seq [12, 20, 31]. Pysam, which functions based on SAMtools, is another approach utilized to explore variations in scRNA-seq data. For instance, Ludwig et al. detected mitochondrial mutations with the pysam module and showed that the allele frequencies estimated from scRNA-seq were consistent with those estimated from whole genome sequencing [17].

Other tools, including FreeBayes [13] and BamBam [32], have also been sporadically used for variant detection in scRNA-seq data, although these tools were originally designed for bulk sequencing and have not been adapted for scRNA-seq data. Notably, BamBam and other callers, like JointSNVMix, Seurat, and SomaticSniper, were not included in our benchmarking, as they require paired normal data to call variants from RNA-seq [33–35].

### Performance evaluation of variant callers on real data

We generated full-length transcriptome data of 291 CD45^−^ single cells with SMART-seq2 protocol. Among these CD45^−^ cells, 70 were identified as malignant cells (Additional file 1), which were derived from two colorectal cancer patients (P0411 and P0413). The average sequencing depths of these cells were 1.4 million reads per cell. Germline single-nucleotide polymorphisms (SNPs) can be identified from bulk exome sequencing (Exome-seq) data and are expected to occur in each of the single cells, and thus, the SNPs detected from bulk Exome-seq can be used as gold standard for single-cell variant calling. Therefore, we also generated bulk WES data of tumor and adjacent normal tissues for these two patients.

To generate gold standard variants from bulk WES data, we aligned reads using the BWA-PICARD pipeline and called SNPs using VarScan2 after filtering out low-quality sequencing reads. To validate the reliability of these SNPs, we further generated bulk RNA-seq data of tumor tissue for patient P0411 and detected SNPs from bulk RNA-seq data by aligning reads with STAR and calling SNPs with SAMtools. We found that of all the 5861 sufficiently expressed (read depths > 5 in RNA-seq data) benchmark SNPs called from bulk WES, 97.8% (5827/5861) could also be independently identified from bulk RNA-seq data, supporting the reliability of our benchmark SNPs.

Genetic variants can be classified into homozygous and heterozygous variants, both of which could provide valuable insights on gene function and could cause pathogenic phenotypes. However, the heterozygous variants might be inconsistent between Exome-seq and RNA-seq data, due to either the lack of sensitivities of the variant-calling methods or the widespread allele-specific expression [36]. Therefore, we mainly focused on homozygous SNPs for benchmarking, unless explicitly stated in certain parts.

**Fig. 1:**
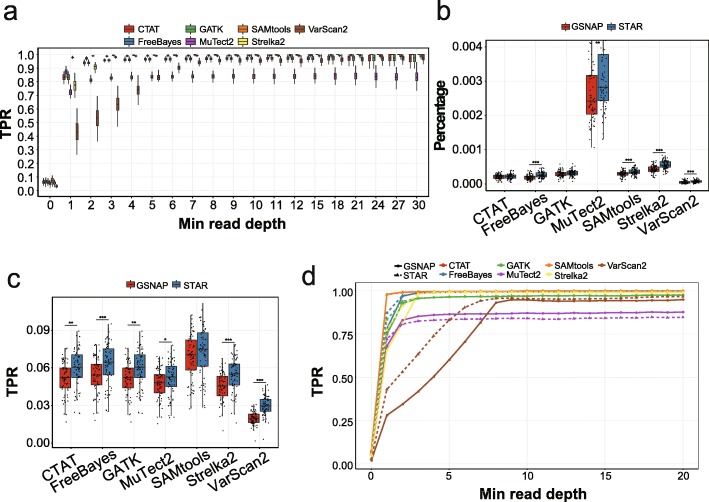
The performance measurements of variant-calling tools in real data. **a** Boxplots showing the TPRs according to the minimal read depths at SNP loci using different variant-calling methods in genome. **b** Boxplots showing the percentages of called variants in ERCC spike-in sequences using two competing aligners. **c** Boxplots showing the TPRs using two competing aligners. **d** Performance curves showing the median TPRs according to the minimal read depths at SNP loci using two competing aligners and different variant calling methods

We used STAR, which was recommended in the GATK pipeline, to align reads from scRNA-seq data. Then, we used the seven variant detection tools to identify SNVs without filtering SNPs and calculated the true-positive rates (TPRs) as proportions of detected variants among the number of benchmark bulk SNPs with a minimal depth. We found that the TPRs were highly dependent on the minimal read depths. The overall sensitivity in median was below 8%; however, SAMtools, FreeBayes, Strelka2, CTAT, and GATK detected more than 95% SNPs in median when read depths were > 2 (Fig. 1a). For most of the tools, the TPRs could reach plateaus with more than two reads, but VarScan2 needed more reads to generate confident results. In general, SAMtools showed the highest sensitivity while MuTect2 and VarScan2 showed lower sensitivity (median values of 82.6% and 63.4%, respectively, with > 2 reads). When restricting to high-confident coding regions, the TPRs were generally higher, but when read depths were increased, they became close to TPRs in whole genome (Additional file 2: Figure S1a). This suggests that sensitivity was associated with genomic contexts, partly attributed to different coverages. Moreover, most variant detection tools could achieve high sensitivity in scRNA-seq data with sufficient read depths.

When analyzing both heterozygous and homozygous SNPs, we found that the overall TPRs decreased as expected due to probably fewer alternative reads. Notably, the relative orders of TPRs for different tools were similar as those based on homozygous SNPs (Additional file 2: Figure S1b).

Synthetic spike-in sequences, which are designed as a standard set of exogenous RNA controls by External RNA Controls Consortium (ERCC), were added into our single-cell libraries before the reverse transcription, and thus, the resulting ERCC variants could serve as negative controls. Our results showed that most of the variant-calling tools, except for MuTect2, identified a median level of less than 0.055% noise variants in the ERCC negative control sequences (Fig. 1b). Notably, VarScan2 identified the fewest ERCC variants, which was expected considering its low sensitivity. Importantly, the averaged read depths of ERCC are much higher than those of RNA reads, which could lead to potential biases when extending the ERCC-based conclusions to real reads (Additional file 2: Figure S1c).

Sequence alignment is an important step in processing short-read data, and unsuitable alignment could dampen the reliable detection of variations. There are several different aligners developed for effective alignment of sequencing data [32], but their performances vary. It is therefore important to assess the capability of individual aligner in terms of performance and accuracy. To compare the impact of aligners on SNV detection, we evaluated STAR and GSNAP, which are commonly used for scRNA-seq data and reported to be reliable general-purpose aligners [32]. We found that the overall TPRs were higher for STAR than GSNAP, especially with low read depths (Fig. 1c, d, Additional file 2: Figure S1d). When reaching plateaus with sufficient read depths, the TPRs for STAR and GSNAP became close. Accordingly, fewer ERCC variants were identified with the GSNAP aligner compared with those identified with the STAR aligner for each variant caller (Fig. 1b).

To make a fair comparison for different tools, we further investigated how the performances of the methods varied based on their key parameters (Additional file 3). For MuTect2, we adjusted the key parameters of log-odds (LOD) threshold (θ_T_) and found that both the sensitivities and the false discovery rates (FDRs) would decrease with higher LOD thresholds. In addition, when the thresholds were reduced to 0, the performance became worse than those with default settings (Additional file 2: Figure S2a, b). For the GATK Best Practices Pipeline, the FDRs would change according to the LOD thresholds, while the sensitivities would not be influenced as much (Additional file 2: Figure S2c, d). We have also adjusted the parameters of VarScan2 and found that both the sensitivities and the FDRs would increase with the adjusted parameters (Additional file 2: Figure S2e, f). Generally, we observed the precision-recall trade-offs. In brief, adjusting parameters were important for SNV-calling tools to achieve best performance, and users should choose the most suitable parameters according to the preference of sensitivities or specificities.

### Evaluation based on simulated data in high-confidence regions

**Fig. 2:**
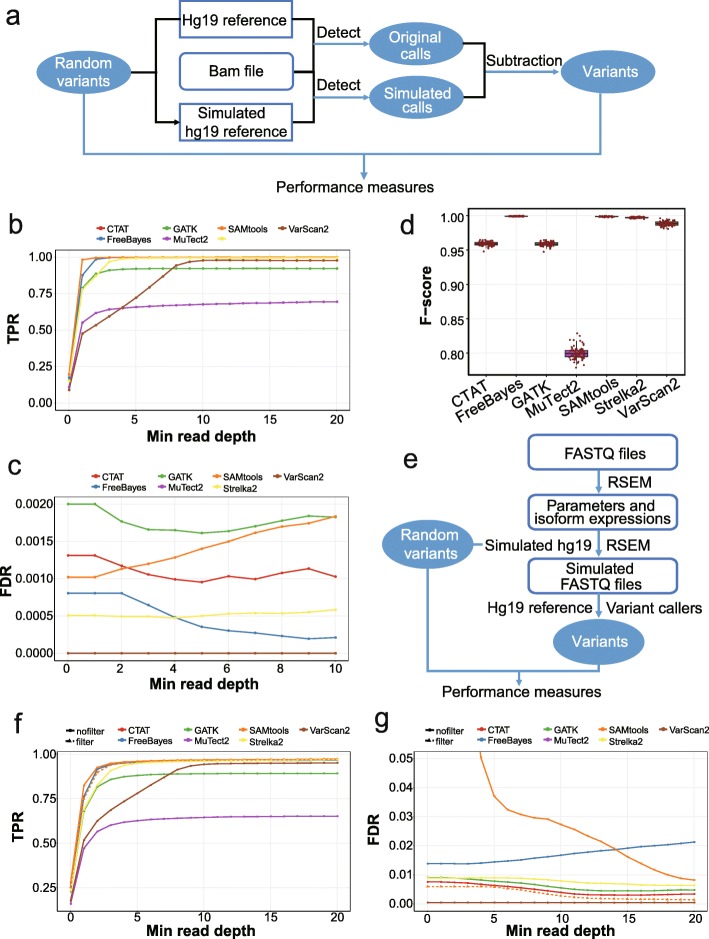
The performance measurements of different variant-calling tools in high-confident coding regions for simulated data. **a** The flowchart showing the simulation procedure of inserting variants into the reference genome and performance assessments. **b**, **c** Performance curves showing the TPRs (**b**) and FDRs (**c**) according to the minimal read depths at inserted SNV loci in high-confident coding regions. **d** Boxplots showing *F*-scores of different tools with read depths ≥ 10. **e** The flowchart showing the simulation procedure based on RSEM. **f**, **g** Performance curves showing the TPRs (**f**) and FDRs (**g**) according to the minimal read depths at inserted SNV loci in high-confident coding regions using RSEM simulation

Simulation is a compelling approach for benchmarking analysis, as the ground truth is known from the process of generating the data, which enables the evaluation of properties of different methods. We thus randomly introduced 50,000 SNVs into the high-confident protein-coding regions of the hg19 reference genome, which represents an ideal genome context, and then compared the called variants of different tools with the expected SNVs (Fig. 2a). The TPRs were calculated as proportions of detections among all expected loci, and the FDRs were defined as proportions of false positives among all detected variants. We recapitulated our results in real datasets that the sensitivity was greatly impacted by read depths and became stable when the minimal read depths were larger than 2, except for VarScan2 (Fig. 2b). Specifically, the median sensitivities for SAMtools, FreeBayes, and Strelka2 reached > 99% with no less than 10 supporting reads, and most tools reached > 92% except for MuTect2. As for the FDRs, the median values were ≤ 0.2% for all tools except for MuTect2, which exhibited a maximal FDR of 2.4% (Fig. 2c). VarScan2 had the highest specificity, followed by Strelka2 and FreeBayes. The GATK-based tool, CTAT, dramatically improved the specificity with no loss of sensitivity compared with GATK. Regarding the *F*-scores with at least 10 reads, FreeBayes, SAMtools, and Strelka2 performed the best with *F*-scores > 0.99 in high-confident coding regions (Fig. 2d). Notably, the overall TPRs calculated based on real data and simulations for each cell were highly correlated (Pearson’s correlation coefficient = 0.958), suggesting the similar performances of SNV-calling tools for the identification of germline SNPs and somatic SNVs in one-sample scRNA-seq analysis.

To further validate our results, we used another simulation method based on RSEM (RNA-Seq by Expectation Maximization) [35]. RSEM, commonly used for RNA-Seq transcript quantification, utilizes a generative model and expectation maximization to estimate isoform expression and is capable of simulating RNA-Seq data based on parameters learned from the real data. We used RSEM and genome reference with spike-in mutations to generate simulated scRNA-seq data with known SNVs, and then compared the spike-in mutations and called variants using the hg19 genome reference (Fig. 2e). We found that the performances of variant callers were highly correlated to those from the first simulation method (Pearson’s correlation coefficient = 0.98 for TPRs and 0.89 for FDRs). The TPRs were quite similar while the FDRs were considerably higher, especially for SAMtools with low read depths (Fig. 2f, g). To investigate the cause of the elevated FDRs, we applied the filter of quality> 20 for SAMtools and found that the FDRs were significantly reduced with similar TPRs. Therefore, low sequencing quality largely influenced the specificity of SAMtools, especially with low read depths. The filter procedure is therefore highly recommended when with low base qualities. Since the first simulation method used real sequencing data, which represent the real distribution of base quality, we applied the first simulation method in the subsequent sections.

### Assessment of the impact of genomic contexts on calling performance

**Fig. 3:**
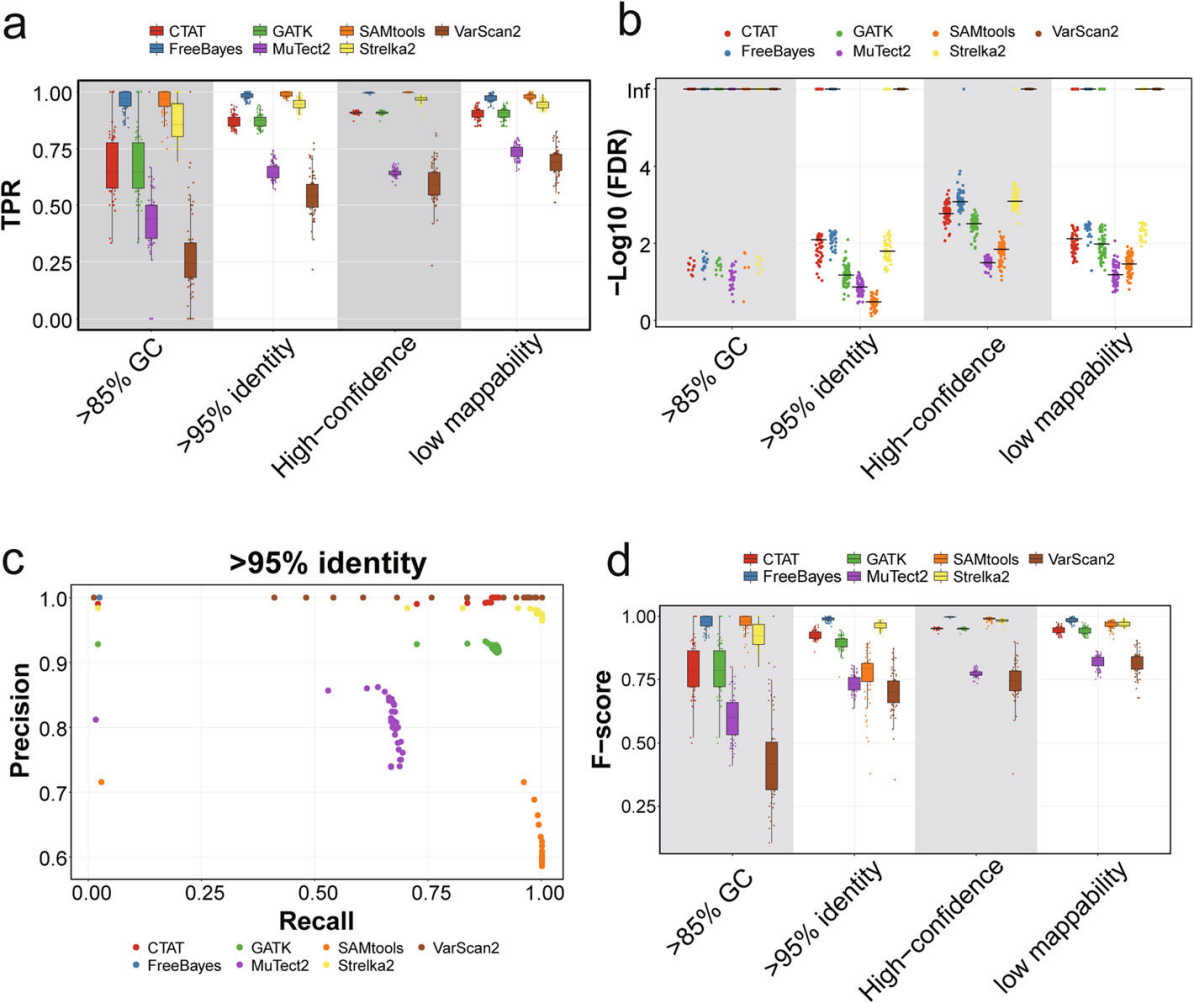
The performance measurements of variant-calling tools in different genomic contexts of simulated data. **a** Boxplots showing the TPRs with minimal read depths ≥ 3 at inserted SNV loci in different genomic contexts. **b** Scatter plots showing the log-transformed FDRs with minimal read depths ≥ 3 at inserted SNV loci. The black lines represent the log-transformed median values of FDRs. **c** Scatter plots showing the precision and recall in different minimal read depth thresholds for high-identity regions. **d** Boxplots showing the *F*-scores with minimal read depths ≥ 3 at inserted SNV loci in different genomic contexts

The genomic contexts could have a great impact on SNV detection for DNA sequencing, as reported by Krusche et al. [37]. We thus used the same classification of genomic regions to investigate the performances of variant-calling methods in different genomic contexts and performed simulations (Fig. 2a). Notably, for regions with high GC content, high sequence identity, or low mappability, the sensitivities were significantly lower than those for high-confidence regions and the variance of TPRs were higher (Fig. 3a, Additional file 2: Figure S3). The order of sensitivities for different tools was similar to that achieved in the high-confident coding regions. SAMtools, FreeBayes, and Strelka2 were the most sensitive tools to different genomic contexts. On the other hand, the FDRs were generally low but higher for the high-identity regions (Fig. 3b, c, Additional file 2: Figure S4). MuTect2 exhibited low accuracy. SAMtools performed generally well but were error-prone in high-identity regions (median FDR = 33.6%). Notably, FreeBayes and Strelka2 performed well with relatively high *F*-scores in different genome contexts (Fig. 3d). In summary, in different genomic contexts, FreeBayes and Strelka2 outperformed other tools in both sensitivities and specificities. SAMtools showed high sensitivities but low specificities especially in high-identity regions.

### Assessment of the impact of functional regions on calling performance

**Fig. 4:**
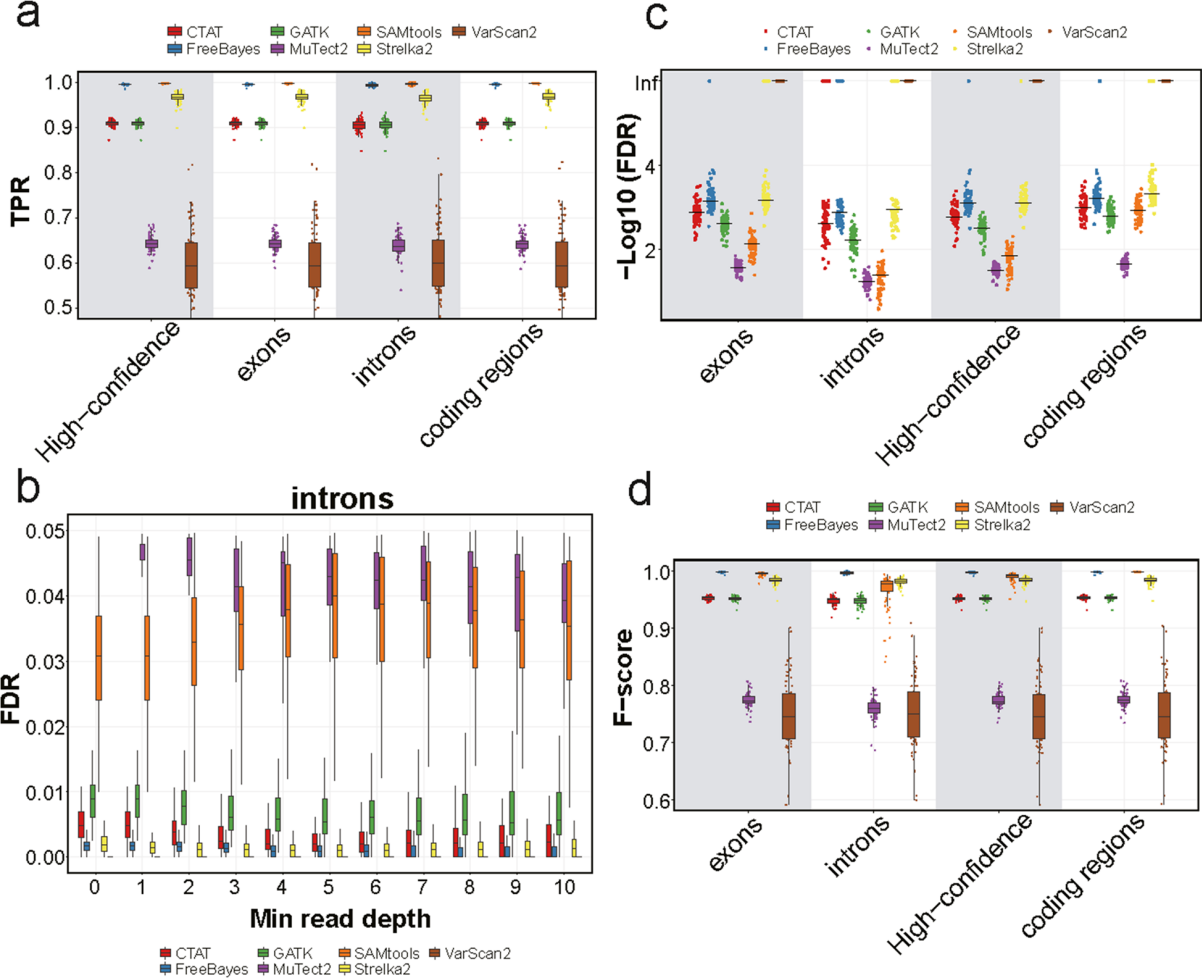
The performance measurements of variant-calling tools in different functional genomic regions of simulated data. **a** Boxplots showing the TPRs with minimal read depths ≥ 3 at inserted SNV loci in high-confidence regions. **b** Boxplots showing the false-positive rates according to the minimal read depths at inserted SNV loci in introns. **c** The scatter plot showing the log-transformed FDRs with minimal read depths ≥ 3 at inserted SNV loci in high-confidence regions. The black lines represent the log-transformed median values of FDRs. The criteria of RefSeq, which we used to annotate coding regions, are more stringent than UCSC, which we annotate exons. Therefore, the performances are slightly different in exons and coding regions. **d** Boxplots showing the *F*-scores with minimal read depths ≥ 3 at inserted SNV loci in high-confidence regions

Next, we restricted our simulations to high-confident regions and investigated the performances of different tools for calling SNVs in exons, coding regions, and introns, as there are still moderate RNA-seq coverages for the intronic regions (Fig. 2a). Although the overall TPRs were much lower for SNVs called in introns than those in exons or in coding regions (Additional file 2: Figure S5a), they become fairly close when restricting minimal read depths to be > 2 (Fig. 4a, Additional file 2: Figure S5b). It suggests that the differences in overall TPRs are mainly because of the lower coverages in introns. Specifically, SAMtools, FreeBayes, and Strelka2 showed the highest sensitivities in all tested functional regions. In contrast, the FDRs did not show relevance to read depths in either introns or coding regions (Figs. 2c and 4b). Median precisions were generally high (> 99%) in introns except for SAMtools (96.4%) and MuTect2 (79.5%) (Fig. 4b, c, Additional file 2: Figure S5). The median *F*-scores in introns with > 2 reads were above 0.9 for the tools except for MuTect2 and VarScan2 (Fig. 4d). Notably, FreeBayes showed the highest *F*-score (0.997 in median) in introns with > 2 reads, followed by Strelka2 (median *F*-score = 0.981). Therefore, FreeBayes and Strelka2 showed superior performances in different functional regions. SAMtools showed highest sensitivity but with low precision in introns (Fig. 4a, c).

### Assessment of the impact of variant allele frequencies on calling performance

**Fig. 5:**
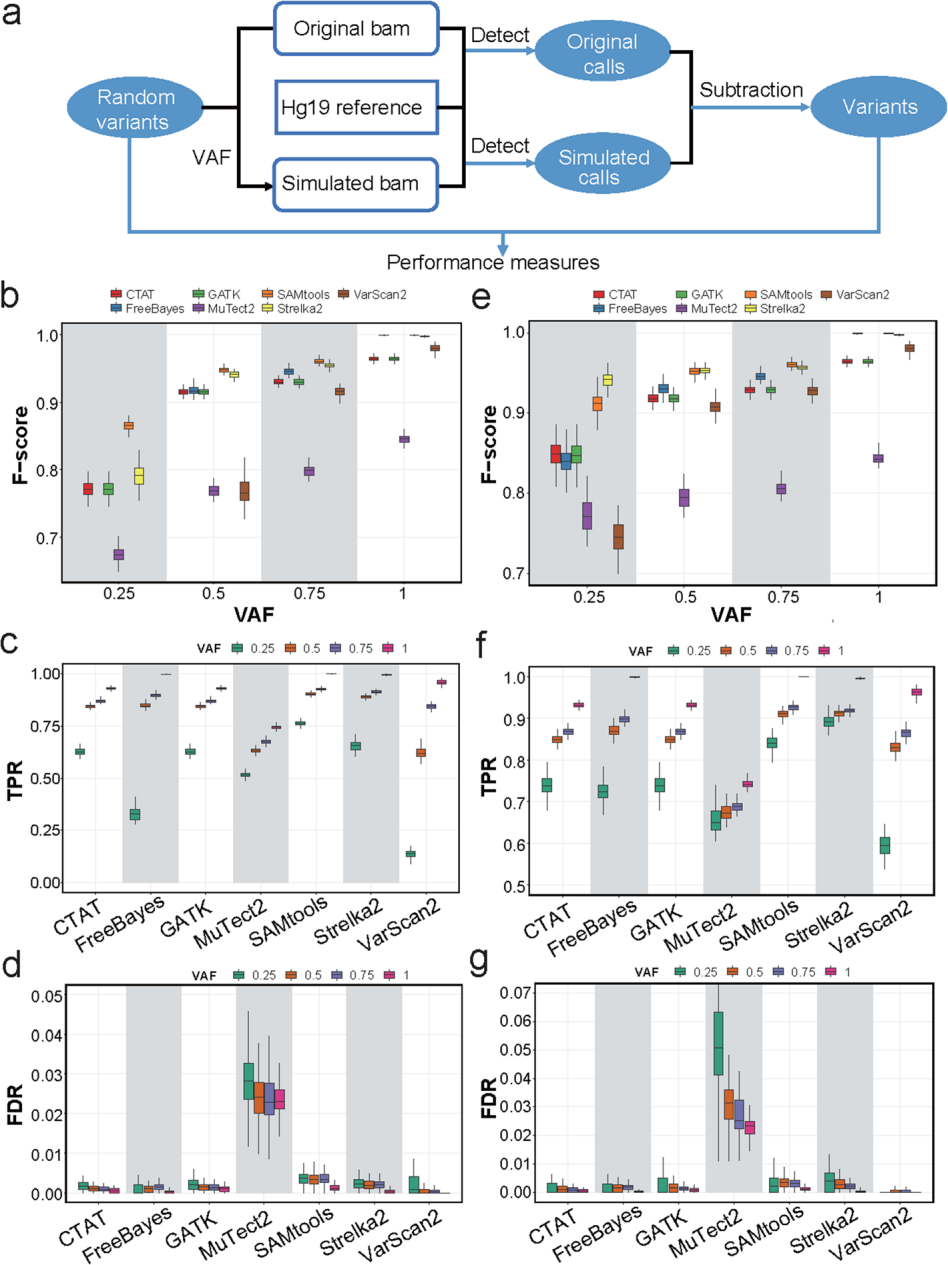
The performance measurements of variant-calling tools with different variant allele frequencies in simulated data. **a** The flowchart showing the simulation procedure of inserting random variants to mapped BAM file and the performance assessments based on simulation. **b**–**d** Boxplots showing the *F*-scores (**b**), TPRs (**c**), and FDRs (**d**) with minimal read depths ≥ 10 at SNP loci in high-confidence coding regions. **e**–**g** Boxplots showing the *F*-scores (**e**), TPRs (**f**), and FDRs (**g**) with minimal supporting reads for the variant ≥ 10 in high-confidence coding regions

In many cases, the variants are heterozygous and both alleles are expressed. We thus investigated the potential influences of variant allele frequencies (VAFs) on the detection performance. We used the BAMSurgeon tool [38] to insert random mutations into the mapped BAM file with different allelic ratios (Fig. 5a). To control the impact of genomic contexts, we restricted all inserted mutations to high-confident coding regions. When requiring the total read depths to be ≥ 10, we observed increasing *F*-scores with higher VAFs (Fig. 5b). SAMtools showed superior performance especially when VAF was low. With the increase of allele frequencies, the performances of different tools became more similar, among which SAMtools and Strelka2 were the best. VarScan2 is the most sensitive tool to VAF, which is concordant to our previous results of its sensitivity to read depths. Regarding the TPRs (Fig. 5c), SAMtools showed the highest sensitivity with a median value of 76.6% for VAF of 25%, 90.3% for VAF of 50%, and 92.6% for VAF of 75%. Notably, FreeBayes showed dramatical decrease of TPRs with low VAFs. As for the precision, the median of FDRs was < 0.5% for all tools except for MuTect2 (Fig. 5d).

Furthermore, we controlled the variant allele read depths to be ≥ 10, a situation in which all of the 7 tools could reach plateaus according to our previous simulation and real data analyses. Indeed, increasing *F*-scores and sensitivities were observed with elevated VAFs (Fig. 5e, f), while FDRs remained low (< 0.5% in median except for MuTect2) at different VAFs for most tools (Fig. 5g). Notably, Strelka2 and SAMtools outperformed other tools regarding *F*-scores, especially when the VAF was particularly low.

### Characteristics of SNVs identified from scRNA-seq data

To characterize the features of SNVs identified from scRNA-seq data, we performed further comparative analyses. We observed substantial sharing of SNVs across multiple cells and also observed non-overlapping distribution of SNVs across cells, suggesting unobserved transcripts or possible heterogeneity among cancerous cells (Additional file 2: Figure S6a–c). In addition, we also compared the number of identified SNVs for all sequenced CD45^−^ cells and found that for all variant-calling methods except for VarScan2, the numbers of SNVs in malignant cells were much higher than those in epithelial cells or fibroblasts (Additional file 2: Figure S6d). The difference might be due to the low sensitivities of VarScan2 with low read depths, for copy number variations, or nonsense-mediated mRNA decay in malignant cells might cause a large number of SNVs to be lowly expressed (Additional file 2: Figure S6e). Furthermore, we found that the proportions of COSMIC mutations in malignant cells were much higher than those in non-malignant cells for all tested tools except for MuTect2 (Additional file 2: Figure S6f).

To delineate the capability of subpopulation identification of the single-cell SNV profiles, we used the Barnes-Hut t-Distributed Stochastic Neighbor Embedding (t-SNE) for dimensionality reduction and performed K-means clustering on the SNV profiles of the 70 malignant cells. The results showed that 70 cells were generally clustered into 2 subpopulations, coordinating to the patient origins. Most tools except for SAMtools could achieve better performances than the clustering result based on gene expression (Additional file 2: Figure S7), suggesting that SNVs could serve as important resource for subpopulation identification. Specifically, VarScan2 achieved the best clustering result (average silhouette width, 0.76) while SAMtools showed the worst (average silhouette width, 0.38). Notably, the clustering methods could be complex and more detailed investigation was needed for further in-depth characterization.

### Performance evaluation of variant callers in different datasets

**Fig. 6:**
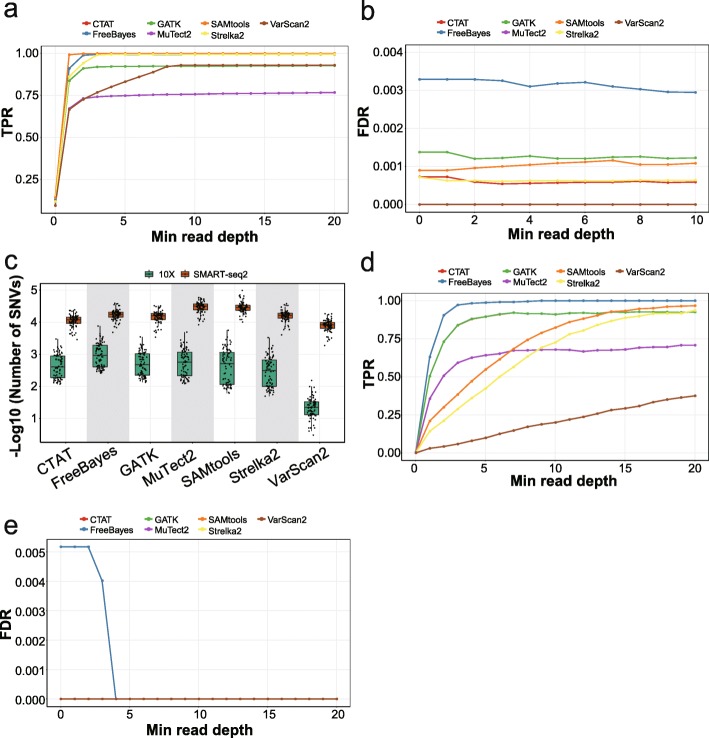
The performance measurements of variant-calling tools in different datasets. **a**, **b** Performance curves showing the median TPRs (**a**) and FDRs (**b**) according to the minimal read depths at SNP loci in high-confident coding regions for the SMART-seq2 hepatocellular carcinoma dataset. **c** Boxplots showing the log-transformed counts of detected SNVs using different sequencing platforms. **d**, **e** Performance curves showing the median TPRs (**d**) and FDRs (**e**) according to the minimal read depths at SNP loci in high-confident coding regions for the 10X hepatocellular carcinoma dataset

To assess the robustness of our benchmark across different datasets, we performed further analysis using scRNA-seq data collected from a patient with hepatocellular carcinoma, as published by Wang et al. [39]. We repeated the aforementioned simulation process (Fig. 2a) on the 77 single cells sequenced by SMART-seq2 and found consistent results with those generated from the 2 colorectal cancer patients. Specifically, SAMtools, Strelka2, and FreeBayes showed the highest median sensitivities of > 99% with no less than 10 reads in high-confidence coding regions (Fig. 6a). The FDRs of FreeBayes increased while the FDRs of VarScan2, Strelka2, CTAT, SAMtools, and GATK remained stable and less than 0.2% (Fig. 6b). In addition, the performances of different variant callers in different genomic regions were also consistent with those for colorectal tumors (Additional file 2: Figures S8 and S9).

10x Genomics Chromium (10X), a widely used single-cell expression profiling platform, allows for the simultaneous transcriptome quantification of a large number of single cells. In spite of the skewness of poly-G enrichment [40] and low coverages, which might limit the application for detecting SNVs, 10X-derived scRNA-seq data could be useful for the investigation of variant-calling performances. Thus, we used different tools to call variants on scRNA-seq data of 78 cells sequenced by 10X in the Wang et al. [39] dataset. As expected, the numbers of detected SNVs were much lower for 10X compared with those for SMART-seq2, despite the same tissue origin of scRNA-seq data (Fig. 6c). We further performed simulation on the 10X dataset and found that the TPRs increased with more supporting reads, which would exceed 90% for most variant callers when reaching stable (Fig. 6d). As for the performances of different SNV-calling tools on 10X data, FreeBayes showed the highest sensitivity, while the precision (with a median value of 99.48%) was lower than other tools (Fig. 6e). In comparison, other tools detected few false SNVs, showing clear precision-recall trade-offs. Our results showed that the overall sensitivities of SNV detection in 10X scRNA-seq data were relatively low, potentially due to the low coverages in vast regions of genome. Notably, the sensitivities increase with high specificities when the supporting read depths increase in high-confidence coding regions.

## Discussion

Single-cell sequencing technologies have profoundly facilitated the understanding of cellular dynamics and have redefined concepts about lineage commitment and development [41]. SNVs could be stably propagated to daughter cells but absent in distantly related cells and thus could serve as intrinsic cellular identifiers [4, 42]. Although scWGS or scWES could be utilized to detect SNVs, such de novo sequencing at single-cell level could be prohibitively expensive and have substantial error rates related to amplification methods, which might hamper the deciphering of cellular dynamics at large scale. Recently, with the innovations of scRNA-seq technology, single-cell transcriptome data have seen explosive growth, forming the potential rich resources for cellular dynamics exploration. Although scRNA-seq has been widely used to characterize the heterogeneity of cell populations, merely measuring gene expression is not enough to evaluate the heterogeneity and lineage relationship of diverse cell types. While gene expression profiling could be subjected to confounding factors and biases that derived from batch effects, cell capture efficiencies, and experimental protocols [43], genetic alterations are associated with such biases in a different manner. Indeed, several studies have explored SNVs in scRNA-seq data to decipher the heterogeneity of cell populations and to track cell lineages retrospectively [12, 17, 44]. Nevertheless, the reliability of such analyses needs to be further evaluated, due to the utility of SNVs detecting tools with different performances, most of which are developed for bulk sequencing data.

Here, we systematically analyzed and compared seven SNV-calling methods on scRNA-seq data. We found that the detection performances of these tools highly depend on the read depths, genomic contexts, functional regions, and variant allele frequencies. When using SMART-seq2, the median sensitivities are above 90% for most tools for homozygous SNVs in high-confidence exons with sufficient read depths (more than 10). However, the sensitivities would decrease when detecting SNVs in regions with high GC content, high identity, or low mappability for all analyzed tools. In addition, low supporting reads and low variant ratios could also reduce the sensitivities. Low read depths could be a result of biologically low expressions or technical bias like dropout events from scRNA-seq. Our results suggest that the improvement of sequencing methods to eliminate dropout events may greatly improve the variant detection effect. The FDRs were generally low (< 1%), which were less impacted by read depths or VAFs compared with sensitivity. Notably, SAMtools, FreeBayes, and Strelka2 achieved the best performance in most situations, among which SAMtools exhibited higher sensitivity but lower specificity, especially when detecting SNVs located in high-identity regions or introns. FreeBayes showed high sensitivities with high VAFs, while the sensitivities decreased with low VAFs, and the specificities were not stable among different datasets. Strelka2 showed stable TPRs and FDRs in different genomic regions and different datasets, while its sensitivities with low read depths were inferior to SAMtools and FreeBayes. In contrast, MuTect2 did not perform well in most cases, which might be because of the lack of matched normal samples. VarScan2 showed the highest specificities, but it needed more supporting reads to generate confident results. Overall, our results highlight the importance of stratification, for example, by genomic contexts or functional regions, in variant calling for scRNA-seq data, which should be noticed in future benchmarking studies and variant-calling applications.

**Fig. 7:**
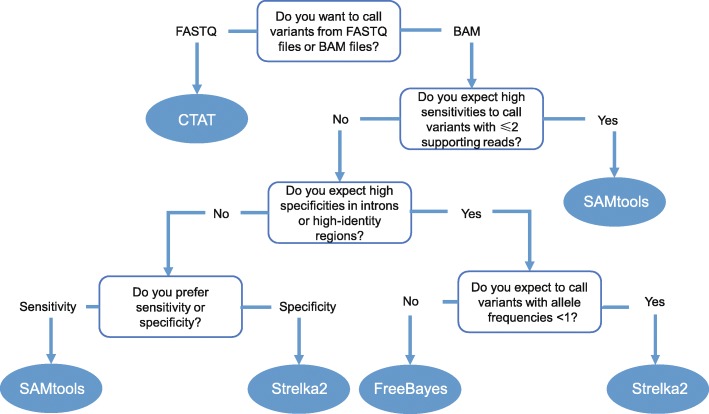
The flowchart demonstrating the recommending process for the choices of suitable SNV-calling methods in scRNA-seq

As for the usability, SAMtools, CTAT, and Strelka2 have advantages. CTAT harbors a built-in aligner and thus has the ability to handle unmapped FASTQ files. Moreover, the alternative parameters enable the flexible usage of CTAT. Besides its superior performances across different genomic regions in our analysis, SAMtools provides clear usage instructions and is user-friendly. In contrast, although pre-processing procedures like sorting and duplicate marking are recommended by FreeBayes, these procedures are not built in the software, thus increasing the difficulty to use. Considering both the performance and usability, we summarize a guideline for the choices of suitable SNV detection tools in different situations when calling variants from scRNA-seq data (Fig. 7). This could serve as a useful reference and shed light on the direction to improving SNV calling in the future.

One possible limitation of our study is that only two aligners, STAR and GSNAP, were compared, as our study mainly focused on the comparison of different variant callers. STAR showed higher sensitivities than GSNAP. More aligners need to be compared further to achieve better performance of variant calling. We showed that parameter adjustment as well as post-filtering could impact the sensitivity or accuracy of variant detection. Detailed comparison of parameter adjustment or post-filtering procedures will further provide insights into the performances of different variant callers in scRNA-seq data. It should also be noticed that the FDR estimations from simulations do not include possible errors introduced during reverse transcription or PCR, although the percentages of variants called in the ERCC spike-in sequences took them into account. Moreover, we performed one-sample analysis to identify SNVs. In this case, we obtained similar results in identifying germline SNPs or somatic SNVs and thus gave same recommendations. However, it could be a different story for analysis with match normal samples and the differences of identifying germline SNPs or somatic SNVs should be noted.

The main limitation of SNV-calling methods in scRNA-seq is the low sensitivity when detecting variants with low read depths or variants with low VAFs in introns or other specific genome contexts. This is especially true for 10x Genomics data. Although identifying SNVs using 10X data could not profile the global landscape of variants, it could still be useful in certain conditions such as lineage tracing. Our analyses showed that there is still room for improvement of the SNV detection from scRNA-seq. On the one hand, the sensitivities are in urgent need to be enhanced; on the other hand, the specificities should be ensured. Due to the great importance of calling SNVs from scRNA-seq data, methods with better performance warrant further investigation.

## Conclusions

Based on a comprehensive benchmark for the applications of seven variant detection tools in scRNA-seq data, we recommend SAMtools, FreeBayes, Strelka2, or CTAT in different conditions to optimally detect SNVs in scRNA-seq data with low read depths, with high variant allele frequencies, and with sufficient supporting reads, or to process FASTQ files, respectively (Fig. 7). We also illustrate the significant influences of read depths, variant allele frequencies, and stratification of genomic regions to the sensitivities of SNV detection in scRNA-seq data. Our results not only provide a guideline for variant detection, but also highlight the necessity of improving detection sensitivity in comprehensive conditions when developing variant-calling methods for scRNA-seq.

## Methods

### Sample collection and single-cell RNA-seq

Two patients were enrolled and pathologically diagnosed with colorectal cancer at Peking University People’s Hospital. This study was approved by the Research and Ethical Committee of Peking University People’s Hospital and complied with all relevant ethical regulations. Written informed consent was provided by these patients. Single cells were collected and sorted from freshly dissected tumors as we described previously [45]. Briefly, tumors were cut into 1 mm^3^ pieces and enzymatically digested with MACS Tumor Dissociation Kit (Miltenyi Biotec), according to the manufacturer’s instruction. The dissociated cells were passed through a 70-μm Cell-Strainer (BD) and centrifuged. After removing the supernatant and lysing the red blood cell (Solarbio), the pelleted cells were re-suspended and stained with antibodies against CD45 (anti-human CD45, HI30, eBioscience) for FACS sorting, performed on a BD Aria III instrument. After FACS analysis, we conducted single-cell transcriptome amplifications according to the SMART-seq2 protocol as we described previously [45, 46]. We added the External RNA Controls Consortium (ERCC, Ambion; 1: 4,000,000) as exogenous spike-in control before the reverse transcription. Multiplex (384-plex) libraries were constructed and amplified using the TruePrep DNA Library Prep Kit V2 for Illumina (Vazyme Biotech). After purification and quality assessment by fragment analyzer, the pooled libraries were analyzed by an Illumina Hiseq 4000 sequencer with 150-bp paired-end reads.

### Bulk DNA and RNA isolation and sequencing

Genomic DNA isolation and bulk DNA sequencing were performed as we described in our previous work [45]. Briefly, fresh tumors were surgically resected from these two patients. Each tissue was cut into two pieces, with one for further single-cell collection and the other for bulk sequencing. This procedure could maximally ensure that the single-cell and bulk sequencing data were generated from a close region of the tissue. Genomic DNA were extracted using the QIAamp DNA Mini Kit (QIAGEN). Exon libraries were constructed using the SureSelectXT Human All Exon V5 capture library (Agilent). Samples were sequenced on the Illumina Hiseq 4000 sequencer with 150-bp paired-end reads.

For bulk RNA analysis, small fragments of tumor tissues were first stored in RNAlater RNA stabilization reagent (QIAGEN) after surgical resection and kept on ice to avoid RNA degradation. RNA of tumor samples were extracted using the RNeasy Mini Kit (QIAGEN) according to the manufacturer’s specification. Libraries were constructed using NEBNext Poly (A) mRNA Magnetic Isolation Module kit (NEB) and NEBNext Ultra RNA Library Prep Kit for Illumina Paired-end Multiplexed Sequencing Library (NEB). Samples were sequenced on the Illumina Hiseq 4000 sequencer with 150-bp paired-end reads.

### Processing of single-cell RNA-seq data for colorectal cancer datasets

The three-step low-quality read-pair filtering was as described in our previous work [45]. Briefly, we filtered the low-quality reads when (1) “N” bases accounting for 10% read length, or (2) bases with quality < 5 account for 50% read length, or (3) containing adapter sequences. The remaining paired-end reads were aligned to the hg19 human genome reference downloaded from UCSC using STAR (2.7.0f_0328) and GSNAP (2011-03-28.v3).

We used the R package tximport (version 1.9.12) to summarize the transcript-level estimated counts into the matrix of gene-level counts.

### Identification of malignant cells

To distinguish malignant cells from non-malignant cells in CD45^−^ cells generated by SMART-seq2, we used t-SNE for dimensionality reduction and performed K-means clustering on all the sequenced CD45^−^ cells. As a result, cells were partitioned into three clusters (Additional file 2: Figure S10a), including fibroblasts, normal epithelial, and malignant cells, each with unique signature genes. Fibroblasts highly expressed classical markers of *ENG*, *COL1A2*, and *ACTA2* (Additional file 2: Figure S10b), while the remaining two clusters were composed of epithelial cells, characterized by the high expression of the Epithelial Cell Adhesion Molecule (*EPCAM*). Notably, one of the two clusters was characterized by specific expression of cell cycle-related genes including *MKI67* and *CDK1*, as well as cancer-associated genes including *S100A14*, *MUC13*, and *KRT7*, and thus was defined as malignant cells (Additional file 2: Figure S10b). In addition, the malignant cell cluster harbored much higher number of expressed genes (Additional file 2: Figure S10c) and showed large-scale chromosomal copy-number variations inferred based on the transcriptome data (Additional file 2: Figure S10d), further confirming the malignant phenotype of this cell cluster.

### Bulk Exome-seq data and RNA-seq data processing

We filtered out low-quality sequencing reads with the same procedure as scRNA-seq data processing. Then, we aligned reads using the BWA-PICARD pipeline and called SNVs using VarScan2 on bulk Exome-seq data. For bulk RNA-seq data, we aligned reads with STAR and called SNVs using SAMtools.

### Variant/mutation-calling programs

GATK (4.1.0.0), FreeBayes, SAMtools/BCFtools (bcftools-1.9), Strelka2 (2.9.10.centos6_x86_64), Mutect2 (gatk-4.0.4.0), CTAT, and VarScan2 (v2.4.3) were evaluated for their performances of variant detection in scRNA-seq samples. We used the default settings to generate a fair comparison, except for the specific part of discussing parameter adjustment. The detailed parameters and procedures were provided in Additional file 3.

### Genomic region stratification

We used Krusche’s definition of region stratification. In brief, the high GC regions were those with > 85% GC adding 50 bp on each side. The repetitive regions were those with > 95% identity adding 5 bp slop. The low mappability regions were generated based on GEM mappability tool, and regions considered difficult to map by amplab SiRen. The high-confidence protein-coding regions were generated by intersection of the Refseq protein-coding regions and GIAB pilot sample NA12878/HG0016 high-confidence regions identified by the Global Alliance for Genomics and Health Benchmarking Team (GA4GH) [37]. We downloaded the bed files in https://github.com/ga4gh/benchmarking-tools. The hg19 introns and exons were downloaded using USCS table browser.

### Evaluation based on bulk sequencing

Although we were not able to evaluate the performance of somatic SNV identification based on bulk sequencing data, because of the heterogeneity for tumors, germline SNPs identified with bulk Exome-seq are expected to exist in each cancer cell. Thus, we calculated TPRs for each cancer cell as the proportion of identified SNPs using scRNA-seq in the number of SNPs detected using bulk Exome-seq.

### Simulation

First, we called variants with one of the competing tools using the hg19 reference. Then, we inserted 50,000 random SNVs into the hg19 reference, restricting them to the targeted regions and avoiding 100 bp around the originally called SNVs for the sample. Then, we called SNVs using the simulated reference, filtering those identified as SNVs using original reference, and compared the derived SNVs with the inserted random variants.

In the RSEM simulation, we first called isoform level expression and calculated the parameters using “rsem-calculate-expression” command. Then, we inserted 50,000 random SNVs into the hg19 reference as above. We simulated FASTQ files with the simulated reference using “rsem-simulate-reads” command, producing 2,500,000 reads per sample. Then, we called SNVs using the original hg19 reference and compared the derived SNVs with the inserted random variants.

To modify the variant allele frequencies, we used BAMSurgeon [38] to insert random variants with VAFs 0.25, 0.5, and 0.75, to the original BAM file. Then, we subtracted variants called with original BAM file from variants called with simulated BAM file and compared the resulting calls with the inserted random variants. Variants inserted in each cell were different in simulation process, representing the somatic SNVs.

We calculated TPRs as the proportion of identified random variants in all the inserted variants for each cell. We calculated FDRs as the proportion of wrong variants among all called variants.

### Variant comparison

We used the RTG Tool vcfeval to compare SNVs with the parameters “--squash-ploidy.”

### Defining sensitivity and specificity

We defined the number of inserted mutations as true and the detected SNVs as positive.

Sensitivity (true-positive rate, recall) = detected inserted mutations/number of inserted mutations

Specificity (precision) = detected inserted mutations / number of detected mutations

False discovery rate = 1 − detected inserted mutations / number of detected mutations

*F*-score = 2 × Specificity × Sensitivity/(Specificity + Sensitivity)

### Processing of scRNA-seq data for liver cancer datasets

For cells sequenced using SMART-seq2, genes expressed (TPM > 0) in less than 10 cells were filtered out. Cells were removed according to the following criteria: (1) cells that had fewer than 800 genes and (2) cells that had over 50% reads mapped to mitochondrial genes. We used GSNAP to align reads.

For cells sequenced using 10X, the alignment was performed by CellRanger (version 2.2) as described by Wang et al. [39].

## Supplementary information

**Additional file 1.** Sequencing statistics of single malignant cells of two colorectal cancer patients.

**Additional file 2.** Supplementary figures.

**Additional file 3.** The detailed description of settings for variant calling methods.

**Additional file 4.** Review history.

## Data Availability

The data that support the findings of this study are available at EGA with accession numbers EGAD00001005373 [49] and EGAD00001005448 [39]. The open source code is available on Github https://github.com/fenglin0/benchmarking_variant_callers [47] and can also be accessed at zenodo 10.5281/zenodo.3491658 [48].

## References

[1] Abbosh C, Birkbak NJ, Wilson GA, Jamal-Hanjani M, Constantin T, Salari R (2017). Phylogenetic ctDNA analysis depicts early stage lung cancer evolution. Nature..

[2] Martincorena I, Raine KM, Gerstung M, Dawson KJ, Haase K, Van Loo P (2017). Universal patterns of selection in cancer and somatic tissues. Cell.

[3] Navin N, Krasnitz A, Rodgers L, Cook K, Meth J, Kendall J (2010). Inferring tumor progression from genomic heterogeneity. Genome Res.

[4] Ju YS, Martincorena I, Gerstung M, Petljak M, Alexandrov LB, Rahbari R (2017). Somatic mutations reveal asymmetric cellular dynamics in the early human embryo. Nature..

[5] Miller CA, White BS, Dees ND, Griffith M, Welch JS, Griffith OL (2014). SciClone: inferring clonal architecture and tracking the spatial and temporal patterns of tumor evolution. PLoS Comput Biol.

[6] Zafar H, Wang Y, Nakhleh L, Navin N, Chen K (2016). Monovar: single-nucleotide variant detection in single cells. Nat Methods.

[7] Ross EM, Markowetz F. OncoNEM: inferring tumor evolution from single-cell sequencing data. Genome Biology. 2016;17:69. 10.1186/s13059-016-0929-9 PMC4832472 27083415

[8] Xu X, Hou Y, Yin X, Bao L, Tang A, Song L (2012). Single-cell exome sequencing reveals single-nucleotide mutation characteristics of a kidney tumor. Cell..

[9] Kester L, van Oudenaarden A (2018). Single-cell transcriptomics meets lineage tracing. Cell Stem Cell.

[10] Chen G, Ning B, Shi T. Single-cell RNA-Seq technologies and related computational data analysis. Front Genet. 2019;10 Available from: https://www.frontiersin.org/articles/10.3389/fgene.2019.00317/full. [cited 2019 May 24]. 10.3389/fgene.2019.00317 PMC6460256 31024627

[11] Hwang B, Lee JH, Bang D (2018). Single-cell RNA sequencing technologies and bioinformatics pipelines. Exp Mol Med.

[12] Rodriguez-Meira A, Buck G, Clark S-A, Povinelli BJ, Alcolea V, Louka E, et al. Unravelling intratumoral heterogeneity through high-sensitivity single-cell mutational analysis and parallel RNA sequencing. Molecular Cell. 2019; Available from: http://www.sciencedirect.com/science/article/pii/S1097276519300097. [cited 2019 Mar 14]. 10.1016/j.molcel.2019.01.009 PMC6436961 30765193

[13] Poirion O, Zhu X, Ching T, Garmire LX (2018). Using single nucleotide variations in single-cell RNA-seq to identify subpopulations and genotype-phenotype linkage. Nat Commun.

[14] Deng Q, Ramskold D, Reinius B, Sandberg R (2014). Single-cell RNA-seq reveals dynamic, random monoallelic gene expression in mammalian cells. Science..

[15] Bryois J, Buil A, Evans DM, Kemp JP, Montgomery SB, Conrad DF (2014). Cis and trans effects of human genomic variants on gene expression. PLoS Genet.

[16] Hu P, Lan H, Xu W, Beyene J, Greenwood CM (2007). Identifying cis- and trans-acting single-nucleotide polymorphisms controlling lymphocyte gene expression in humans. BMC Proc.

[17] Ludwig LS, Lareau CA, Ulirsch JC, Christian E, Muus C, Li LH (2019). Lineage tracing in humans enabled by mitochondrial mutations and single-cell genomics. Cell.

[18] Fan J, Lee H-O, Lee S, Ryu D, Lee S, Xue C (2018). Linking transcriptional and genetic tumor heterogeneity through allele analysis of single-cell RNA-seq data. Genome Res.

[19] Enge M, Arda HE, Mignardi M, Beausang J, Bottino R, Kim SK (2017). Single-cell analysis of human pancreas reveals transcriptional signatures of aging and somatic mutation patterns. Cell.

[20] Tirosh I, Venteicher AS, Hebert C, Escalante LE, Patel AP, Yizhak K (2016). Single-cell RNA-seq supports a developmental hierarchy in human oligodendroglioma. Nature..

[21] Quinn EM, Cormican P, Kenny EM, Hill M, Anney R, Gill M (2013). Development of strategies for SNP detection in RNA-seq data: application to lymphoblastoid cell lines and evaluation using 1000 Genomes data. PLoS One.

[22] McKenna A, Hanna M, Banks E, Sivachenko A, Cibulskis K, Kernytsky A (2010). The Genome Analysis Toolkit: a MapReduce framework for analyzing next-generation DNA sequencing data. Genome Res.

[23] Dobin A, Davis CA, Schlesinger F, Drenkow J, Zaleski C, Jha S (2013). STAR: ultrafast universal RNA-seq aligner. Bioinformatics..

[24] Wu TD, Reeder J, Lawrence M, Becker G, Brauer MJ (2016). GMAP and GSNAP for genomic sequence alignment: enhancements to speed, accuracy, and functionality. Methods Mol Biol.

[25] Boutros PC, Ewing AD, Ellrott K, Norman TC, Dang KK, Hu Y (2014). Global optimization of somatic variant identification in cancer genomes with a global community challenge. Nat Genet.

[26] Cibulskis K, Lawrence MS, Carter SL, Sivachenko A, Jaffe D, Sougnez C (2013). Sensitive detection of somatic point mutations in impure and heterogeneous cancer samples. Nat Biotechnol.

[27] Kim S, Scheffler K, Halpern AL, Bekritsky MA, Noh E, Källberg M (2018). Strelka2: fast and accurate calling of germline and somatic variants. Nat Methods.

[28] Koboldt DC, Zhang Q, Larson DE, Shen D, McLellan MD, Lin L (2012). VarScan 2: somatic mutation and copy number alteration discovery in cancer by exome sequencing. Genome Res.

[29] Van der Auwera GA, Carneiro MO, Hartl C, Poplin R, Del Angel G, Levy-Moonshine A (2013). From FastQ data to high confidence variant calls: the Genome Analysis Toolkit best practices pipeline. Curr Protoc Bioinformatics.

[30] Fasterius E, Uhlén M, Szigyarto CA-K (2019). Single-cell RNA-seq variant analysis for exploration of genetic heterogeneity in cancer. Sci Rep.

[31] Li H, Handsaker B, Wysoker A, Fennell T, Ruan J, Homer N (2009). The sequence alignment/map format and SAMtools. Bioinformatics..

[32] Lee M-CW, Lopez-Diaz FJ, Khan SY, Tariq MA, Dayn Y, Vaske CJ (2014). Single-cell analyses of transcriptional heterogeneity during drug tolerance transition in cancer cells by RNA sequencing. PNAS..

[33] Roth A, Ding J, Morin R, Crisan A, Ha G, Giuliany R (2012). JointSNVMix: a probabilistic model for accurate detection of somatic mutations in normal/tumour paired next-generation sequencing data. Bioinformatics..

[34] Christoforides A, Carpten JD, Weiss GJ, Demeure MJ, Von Hoff DD, Craig DW (2013). Identification of somatic mutations in cancer through Bayesian-based analysis of sequenced genome pairs. BMC Genomics.

[35] Larson DE, Harris CC, Chen K, Koboldt DC, Abbott TE, Dooling DJ (2012). SomaticSniper: identification of somatic point mutations in whole genome sequencing data. Bioinformatics..

[36] Borel C, Ferreira PG, Santoni F, Delaneau O, Fort A, Popadin KY (2015). Biased allelic expression in human primary fibroblast single cells. Am J Hum Genet.

[37] Krusche P, Trigg L, Boutros PC, Mason CE, De La Vega FM, Moore BL, et al. Best practices for benchmarking germline small-variant calls in human genomes. Nat Biotechnol. 2019;37:555–60. 10.1038/s41587-019-0054-x PMC6699627 30858580

[38] Ewing AD, Houlahan KE, Hu Y, Ellrott K, Caloian C, Yamaguchi TN (2015). Combining tumor genome simulation with crowdsourcing to benchmark somatic single-nucleotide-variant detection. Nat Methods.

[39] Wang X, He Y, Zhang Q, Ren X, Zhang Z. Direct Comparative Analysis of 10X Genomics Chromium and Smart-seq2. bioRxiv. 2019;615013. https://www.biorxiv.org/content/10.1101/615013v1. 10.1016/j.gpb.2020.02.005 PMC8602399 33662621

[40] Zhang X, Li T, Liu F, Chen Y, Yao J, Li Z (2019). Comparative analysis of droplet-based ultra-high-throughput single-cell RNA-seq systems. Molecular Cell.

[41] Giladi A, Amit I (2018). Single-cell genomics: a stepping stone for future immunology discoveries. Cell..

[42] Lodato MA, Woodworth MB, Lee S, Evrony GD, Mehta BK, Karger A (2015). Somatic mutation in single human neurons tracks developmental and transcriptional history. Science..

[43] Kolodziejczyk AA, Kim JK, Svensson V, Marioni JC, Teichmann SA (2015). The technology and biology of single-cell RNA sequencing. Mol Cell.

[44] Ding J, Lin C, Bar-Joseph Z. Cell lineage inference from SNP and scRNA-Seq data. Nucleic Acids Res. 2019; Available from: https://academic.oup.com/nar/advance-article/doi/10.1093/nar/gkz146/5367412. [cited 2019 Apr 21]. 10.1093/nar/gkz146 PMC6547431 30820578

[45] Zhang L, Yu X, Zheng L, Zhang Y, Li Y, Fang Q (2018). Lineage tracking reveals dynamic relationships of T cells in colorectal cancer. Nature..

[46] Zheng C, Zheng L, Yoo J-K, Guo H, Zhang Y, Guo X (2017). Landscape of infiltrating T cells in liver cancer revealed by single-cell sequencing. Cell.

[47] Liu F, Zhang Y, Zhang L, Li Z, Fang Q, Gao R, Zhang Z. benchmarking_variant_callers. GitHub. 2019; https://github.com/fenglin0/benchmarking_variant_callers. Accessed 23 Oct 2019.

[48] Liu F, Zhang Y, Zhang L, Li Z, Fang Q, Gao R, Zhang Z. Systematic comparative analysis of single-nucleotide variant detection methods from single-cell RNA sequencing data. Zenodo. 2019. 10.5281/zenodo.3491658. 10.1186/s13059-019-1863-4 PMC6862814 31744515

[49] Fenglin Liu, Yuanyuan Zhang, Lei Zhang, Ziyi Li, Qiao Fang, Ranran Gao, & Zemin Zhang. Systematic comparative analysis of single-nucleotide variant detection methods from single-cell RNA sequencing data. Datasets. European Genome-phenome Archive. 2019. https://ega-archive.org/datasets/EGAD00001005373. Accessed 23 Oct 2019. 10.1186/s13059-019-1863-4 PMC6862814 31744515

