# Supplementary material for: Systematic comparative analysis of single-nucleotide variant detection methods from single-cell RNA sequencing data
Source: Genome Biol. 2019 Nov 19;20:242. doi: 10.1186/s13059-019-1863-4 (PMC6862814; doi:10.1186/s13059-019-1863-4)
Supplement: Supplementary file 2 — Additional file 2. Supplementary figures. [file 13059_2019_1863_MOESM2_ESM.pdf]

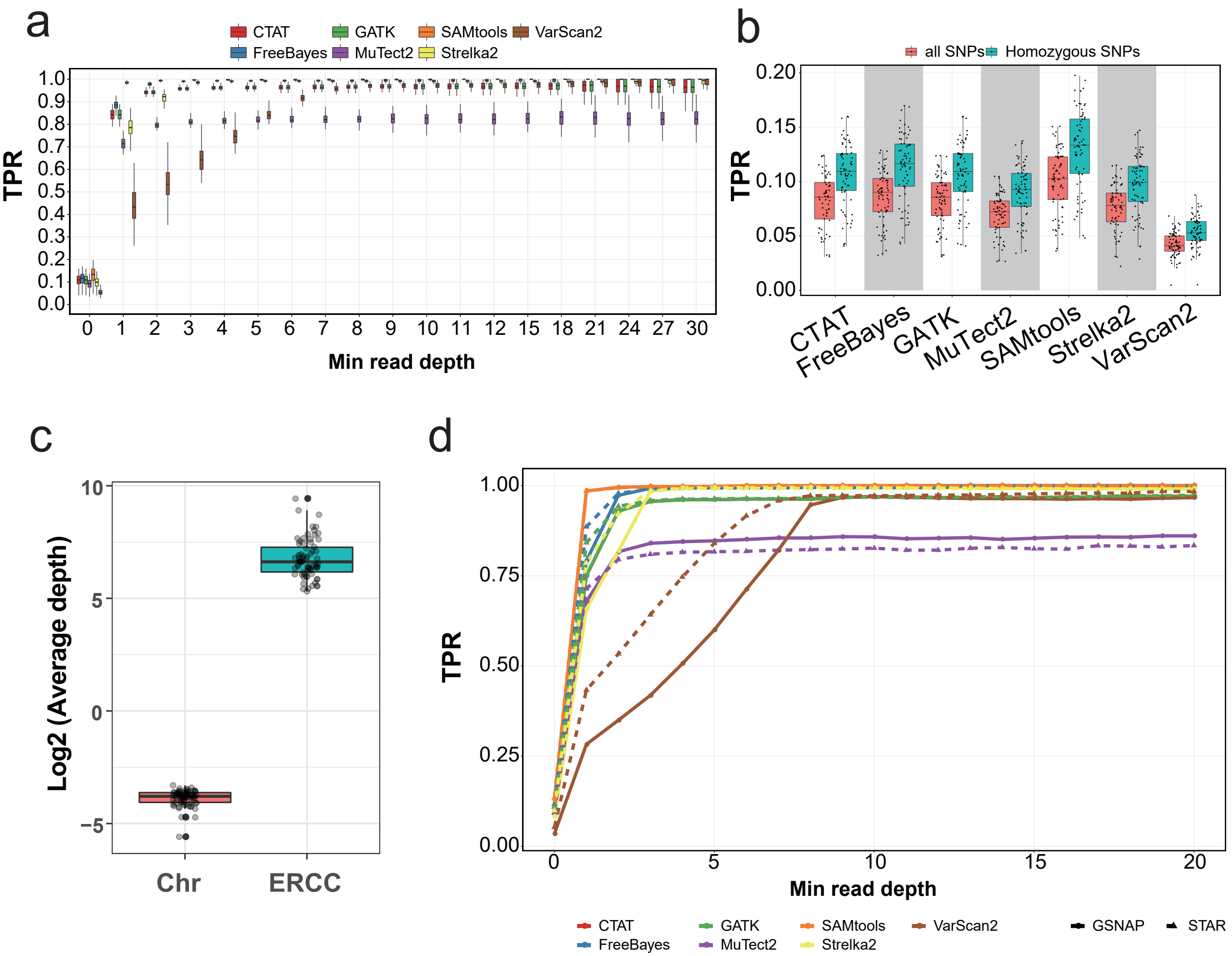

Supplementary Figure 1. The performance measurements of variant-calling tools in real data. (a) Boxplots showing the TPRs according to the minimal read depths at SNP loci using different variant-calling methods in high-confident coding regions. (b) Boxplots showing the overall TPRs for all SNPs or homozygous SNPs. (c) Boxplots showing the average read depths for ERCC and real reads. (d) Performance curves showing the median TPRs according to the minimal read depths at SNP loci in high-confidence coding regions using two competing aligners and different variant calling methods.

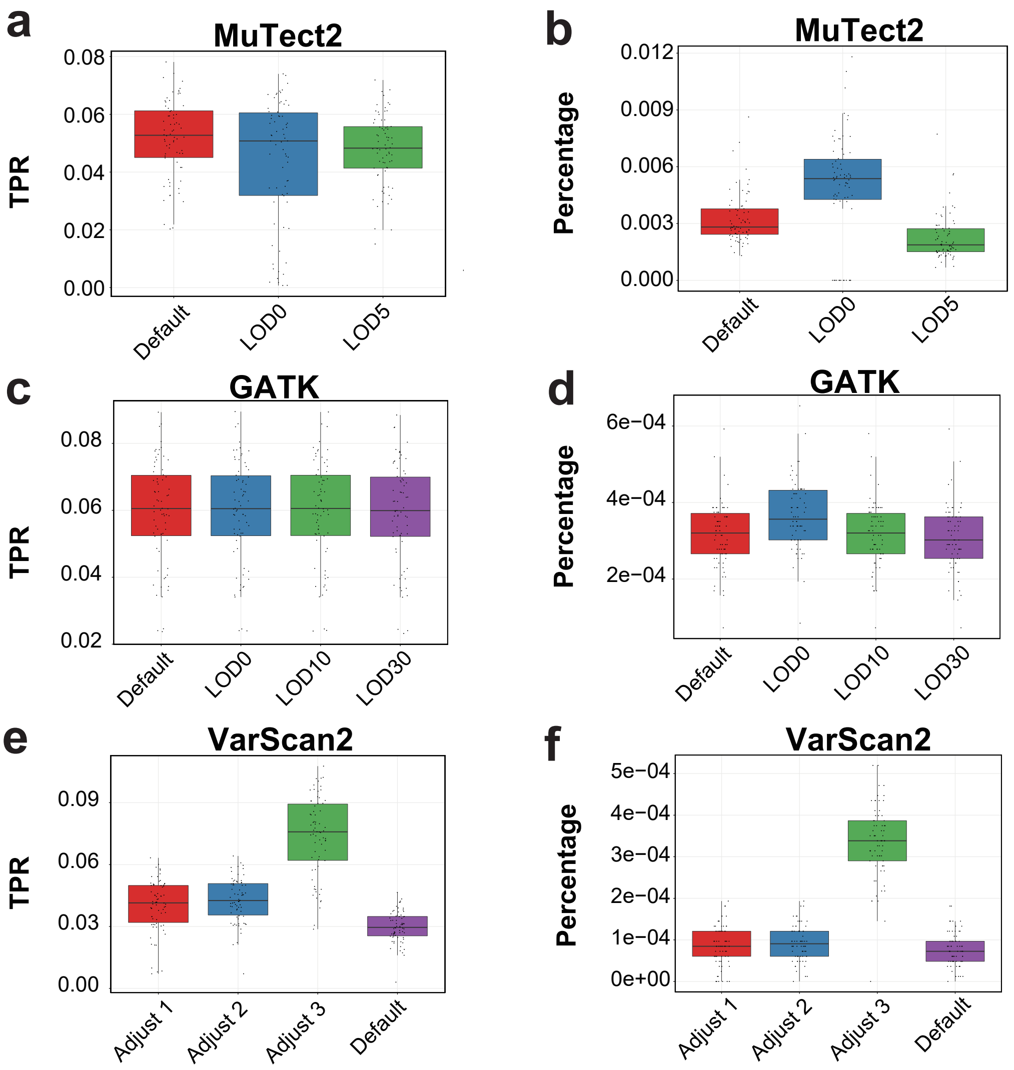

Supplementary Figure 2. The performance measurements of variant-calling tools with adjusted key parameters in real data. (a b) Boxplots showing the TPRs (a) and the percentages of called variants in ERCC spike-in sequences (b) with adjusted key parameters of MuTect2. (c d) Boxplots showing the TPRs (c) and the percentages of called variants in ERCC spike-in sequences (d) with adjusted key parameters of GATK. (e f) Boxplots showing the TPRs (e) and the percentages of called variants in ERCC spike-in sequences (f) with adjusted key parameters of VarScan2. 'Adjust 1', 'Adjust 2' and 'adjust 3' represent the parameters of '--min-coverage 1 --min-reads2 1', '--min-coverage 1 --min-reads2 1 --strand-filter 0', and '--min-coverage 1 --min-reads2 1 --strand-filter 0 --p-value 0.95'.

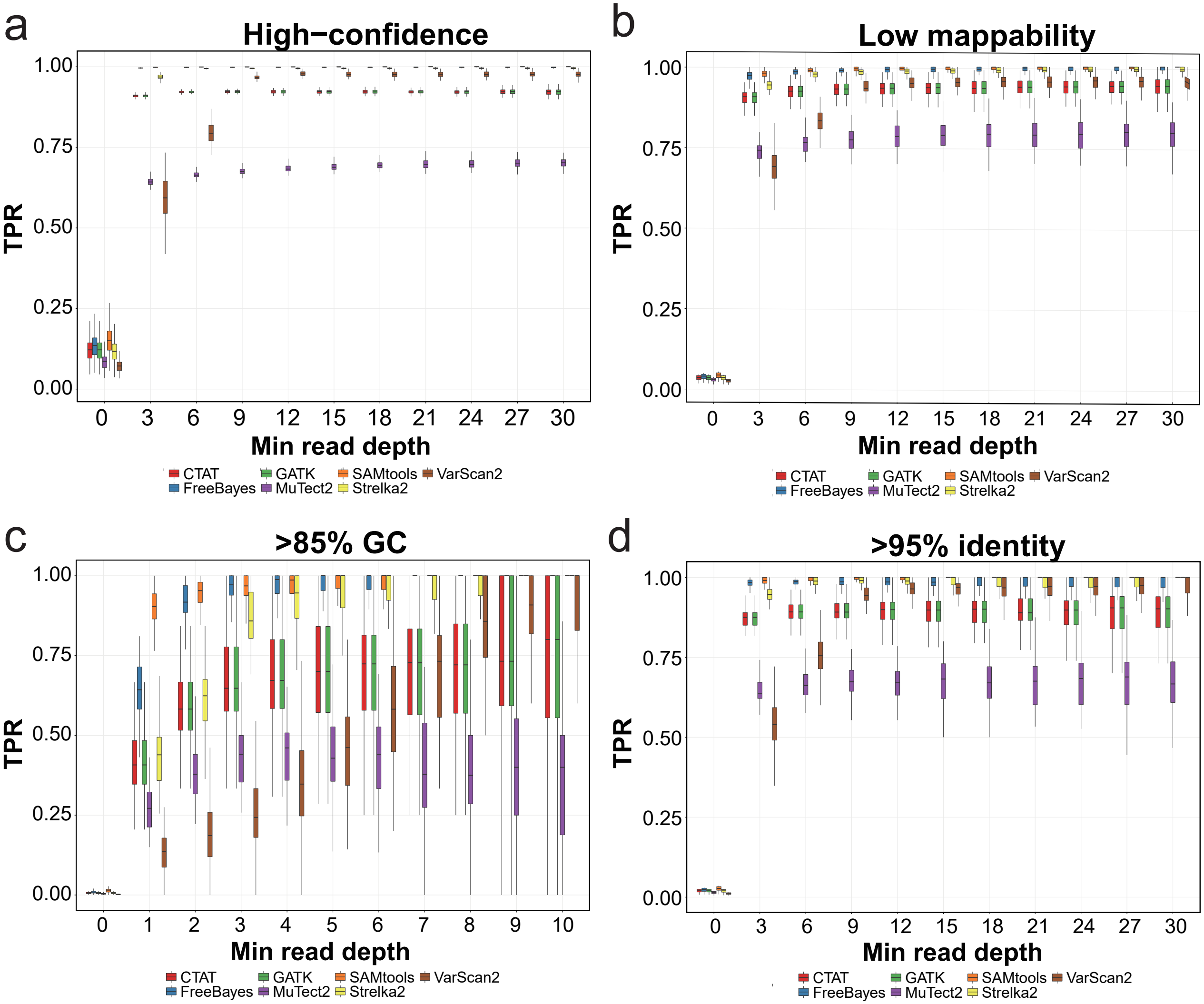

Supplementary Figure 3. The Sensitivities impacted by read depths in different genomic contexts. Boxplots showing the TPRs according to the minimal read depths at inserted SNV loci in high-confidence regions (a), low-mappability regions (b), high-GC regions (c) and high-identity regions (d).

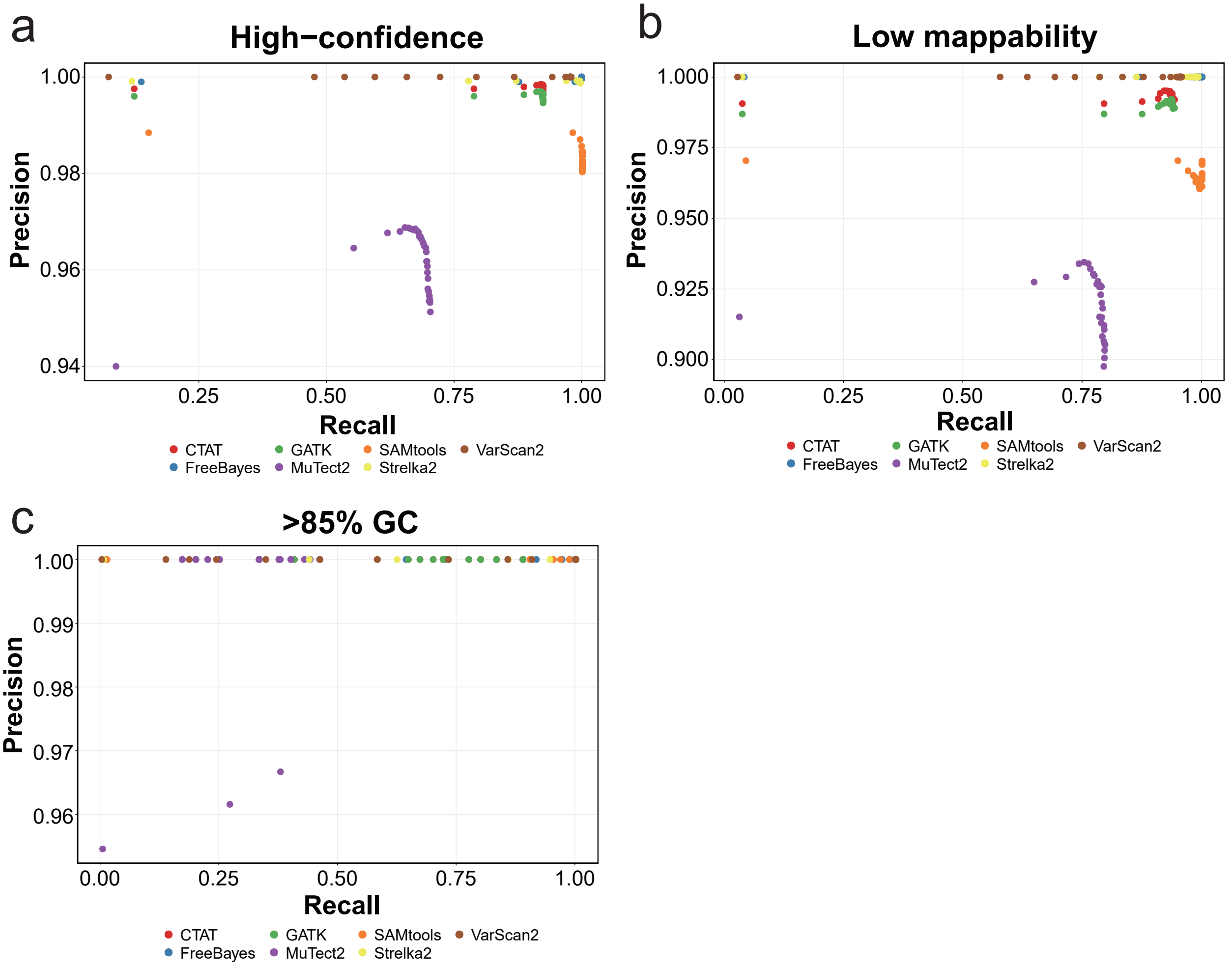

Supplementary Figure 4. The performance measurements of multiple variant-calling tools in different genomic contexts. Scatter plots showing the precision and recall in different minimal read depth thresholds for high-confidence regions (a), low-mappability regions (b) and high-GC regions (c).

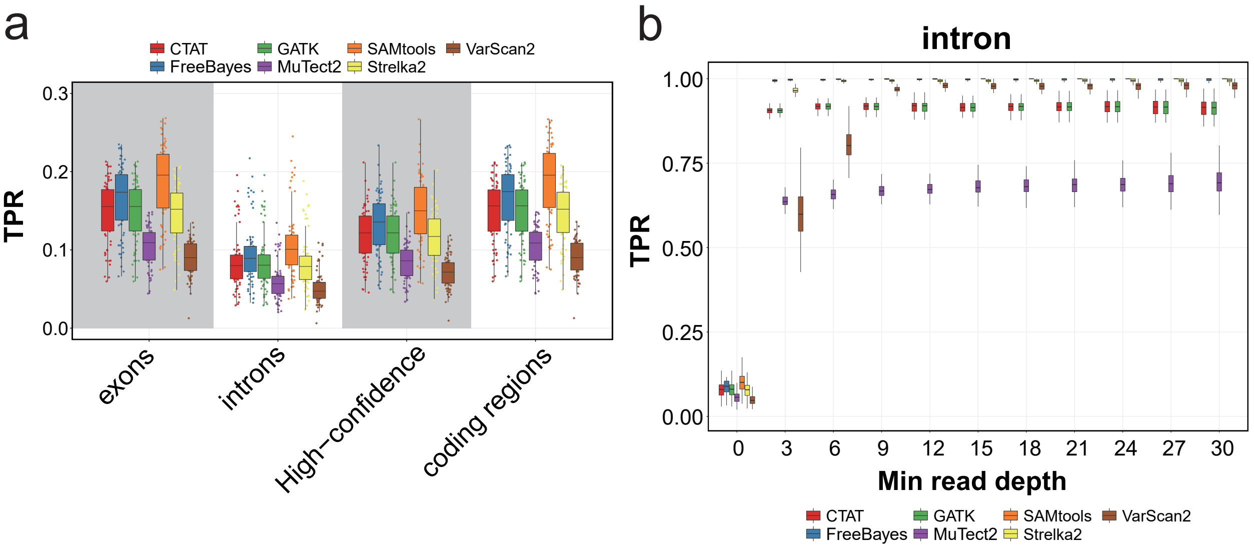

Supplementary Figure 5. The performance measurements of multiple variant-calling tools in different functional regions. (a) Boxplots showing the overall TPRs for variant callers in different functional regions. (b) Boxplots showing the TPRs according to the minimal read depths at inserted SNV loci in introns.

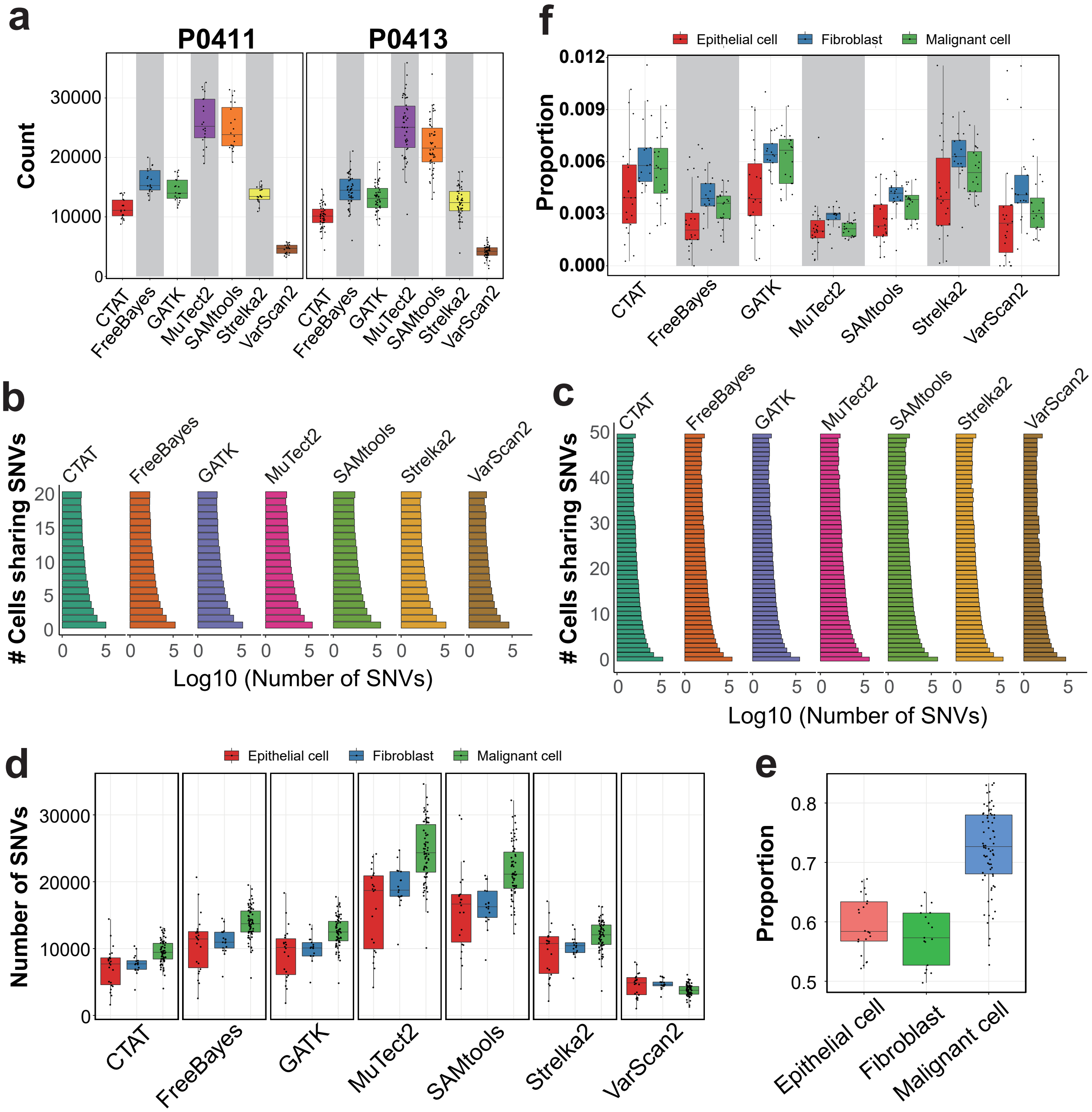

Supplementary Figure 6. Characteristics of SNVs identified by multiple variant-calling tools. (a) Boxplots showing the number of identified SNVs for each patient. (b c) Barplots showing the number of shared SNVs for cells in patient P0411 (b) and P0413 (c). (d) Boxplots showing the number of SNVs identified in different types of cells. (e) Boxplots showing the proportion of variants with low read depths (read depths < 10). (f) Boxplots showing the proportion of COSMIC variants in all SNVs identified by different tools.

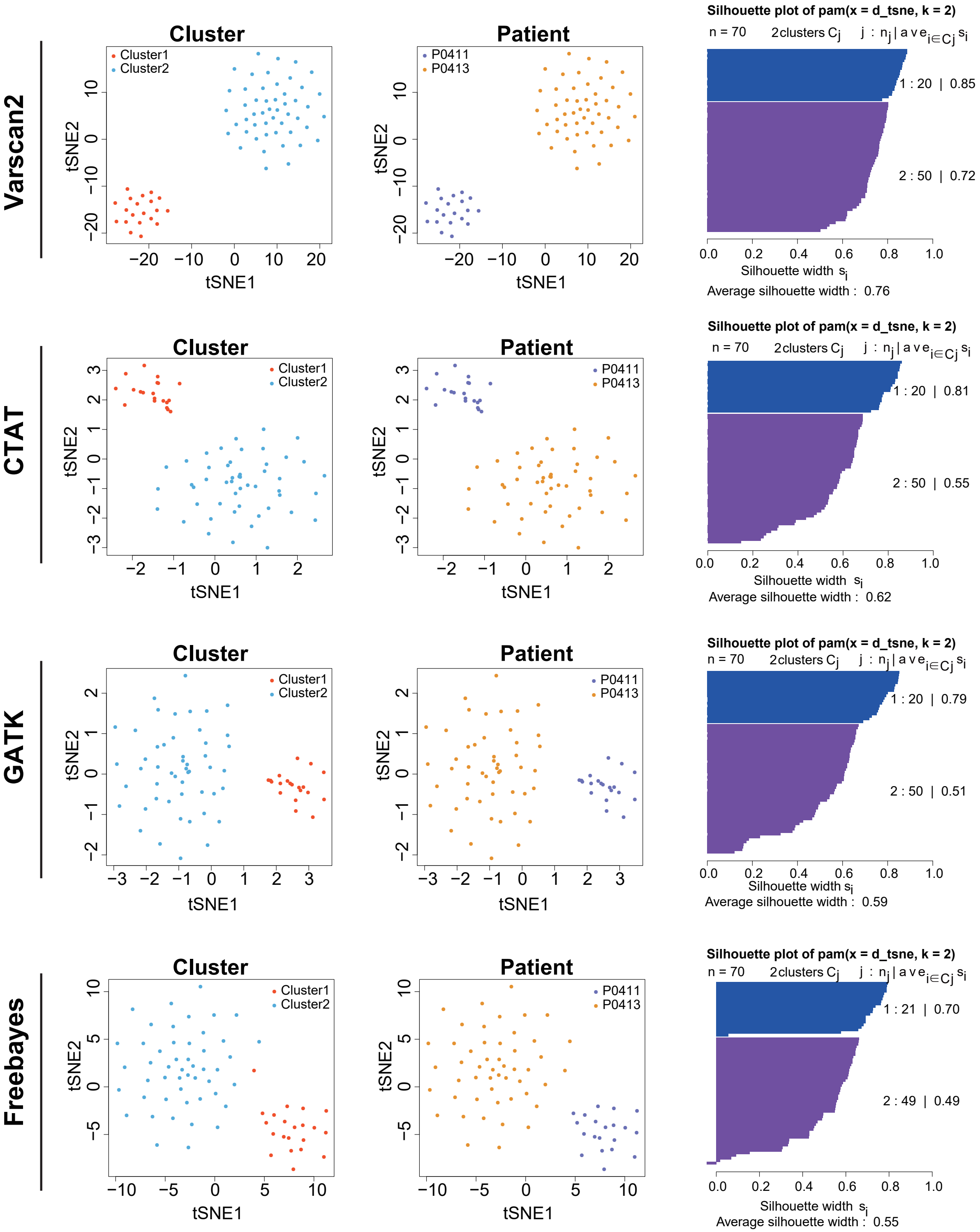

Varscan2
Cluster
Cluster1
Cluster2
Patient
P0411
P0413
tSNE1
tSNE2
Silhouette plot of pam(x = d_tsne, k = 2)
n = 70
2 clusters C_j
$j : n_j | ave_{i \in C_j} s_i$
1 : 20 | 0.85
2 : 50 | 0.72
Silhouette width s_i
Average silhouette width : 0.76
CTAT
Cluster
Cluster1
Cluster2
Patient
P0411
P0413
tSNE1
tSNE2
Silhouette plot of pam(x = d_tsne, k = 2)
n = 70
2 clusters C_j
$j : n_j | ave_{i \in C_j} s_i$
1 : 20 | 0.81
2 : 50 | 0.55
Silhouette width s_i
Average silhouette width : 0.62
GATK
Cluster
Cluster1
Cluster2
Patient
P0411
P0413
tSNE1
tSNE2
Silhouette plot of pam(x = d_tsne, k = 2)
n = 70
2 clusters C_j
$j : n_j | ave_{i \in C_j} s_i$
1 : 20 | 0.79
2 : 50 | 0.51
Silhouette width s_i
Average silhouette width : 0.59
Freebayes
Cluster
Cluster1
Cluster2
Patient
P0411
P0413
tSNE1
tSNE2
Silhouette plot of pam(x = d_tsne, k = 2)
n = 70
2 clusters C_j
$j : n_j | ave_{i \in C_j} s_i$
1 : 21 | 0.70
2 : 49 | 0.49
Silhouette width s_i
Average silhouette width : 0.55

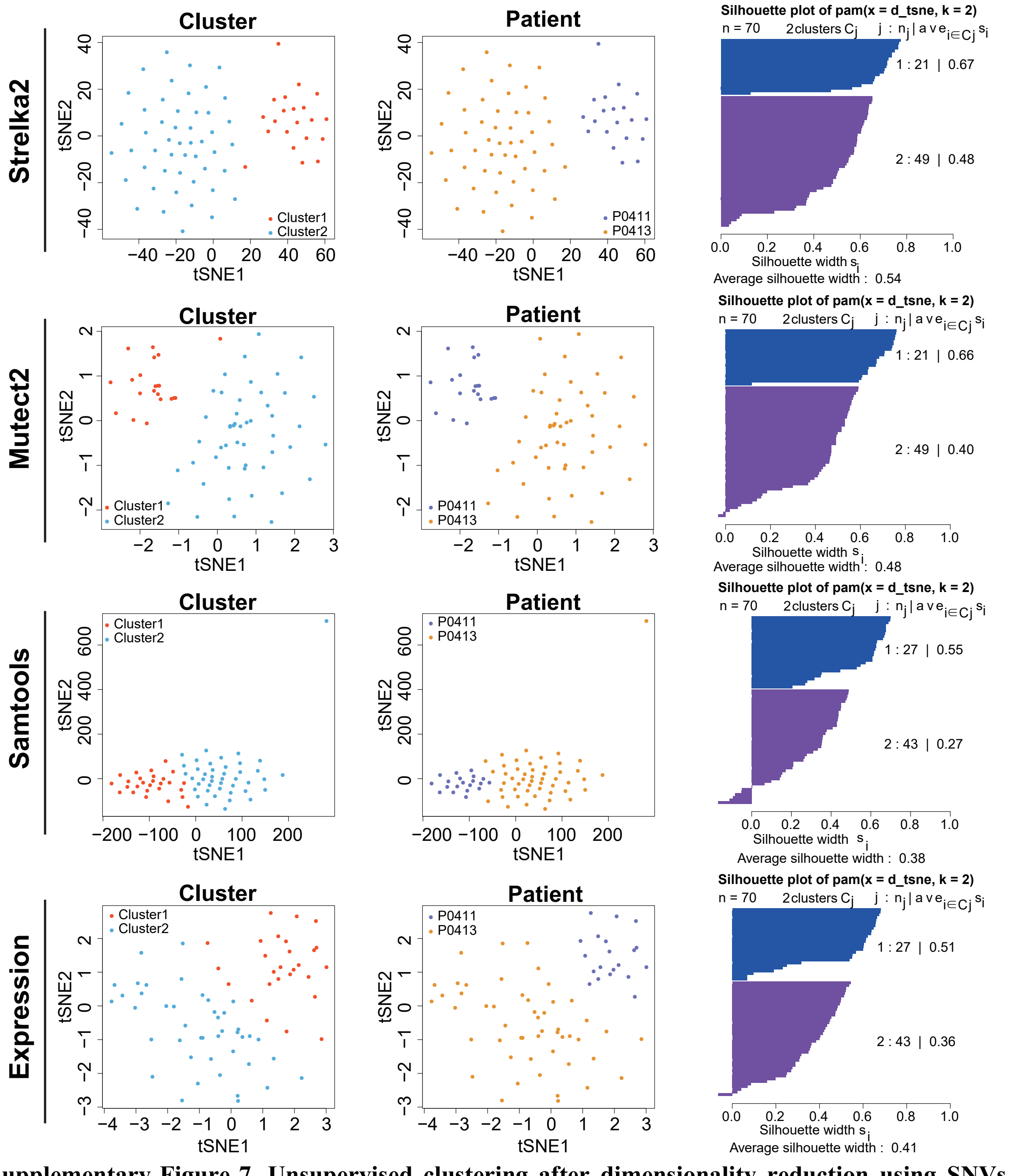

Supplementary Figure 7. Unsupervised clustering after dimensionality reduction using SNVs called by different variant-calling tools compared with the clustering result of transcriptome data. In each method, samples were partitioned into two clusters around medoids on t-SNE map (left) and annotated with patient origins (middle). Silhouette coefficients were used to demonstrate the separation distance between the resulting clusters (right).

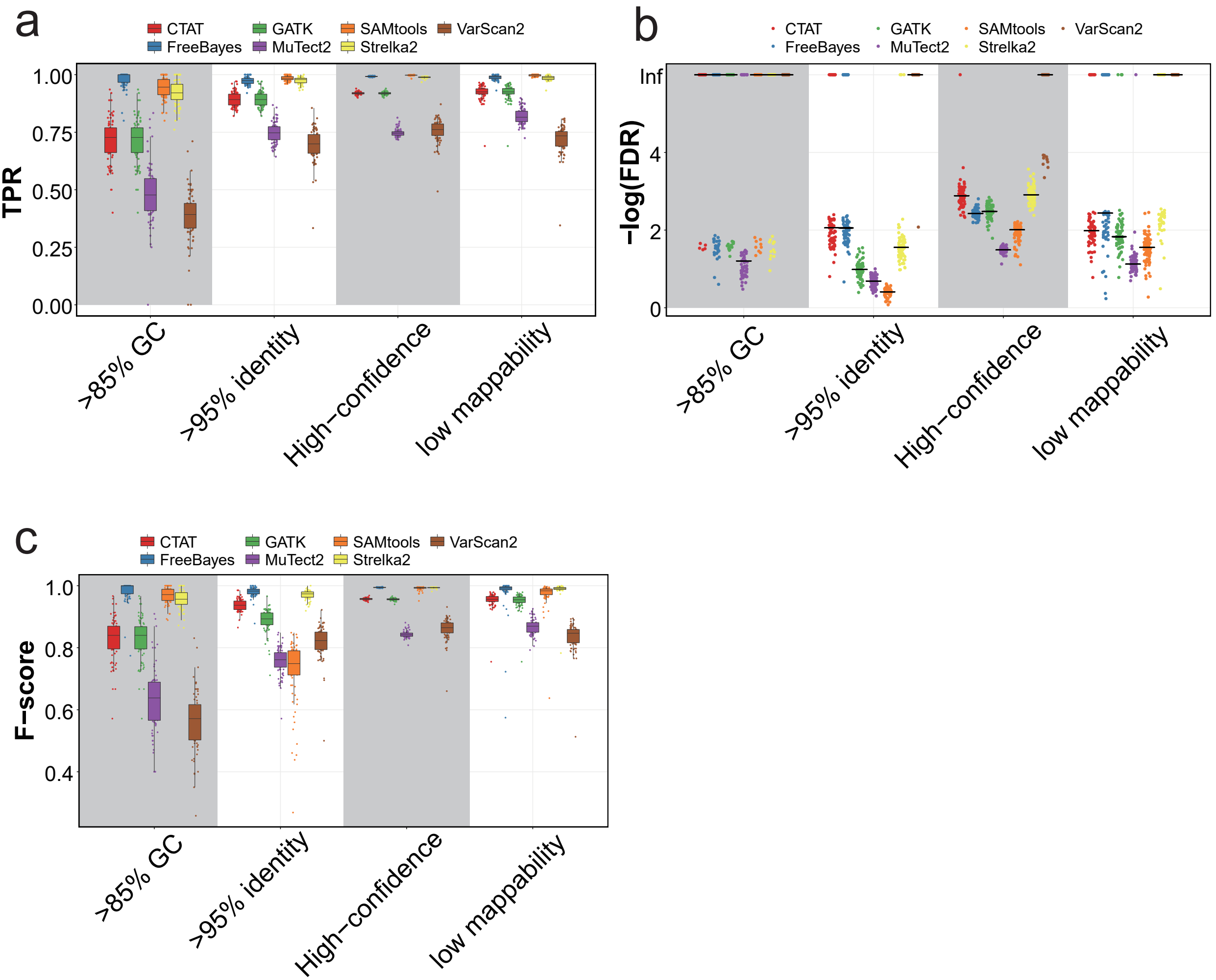

Supplementary Figure 8. The performance measurements of variant-calling tools in different genomic contexts for simulations based on the SMART-seq2 hepatocellular carcinoma dataset. (a) Boxplots showing the TPRs with minimal read depths ≥ 3 at inserted SNV loci in different genomic contexts. (b) Scatter plot showing the log-transformed FDRs with minimal read depths ≥ 3 at inserted SNV loci in different genomic contexts. The black lines represent the log-transformed median values of FDRs. (c) Boxplots showing the F scores with minimal read depths ≥ 3 at inserted SNV loci in different genomic contexts.

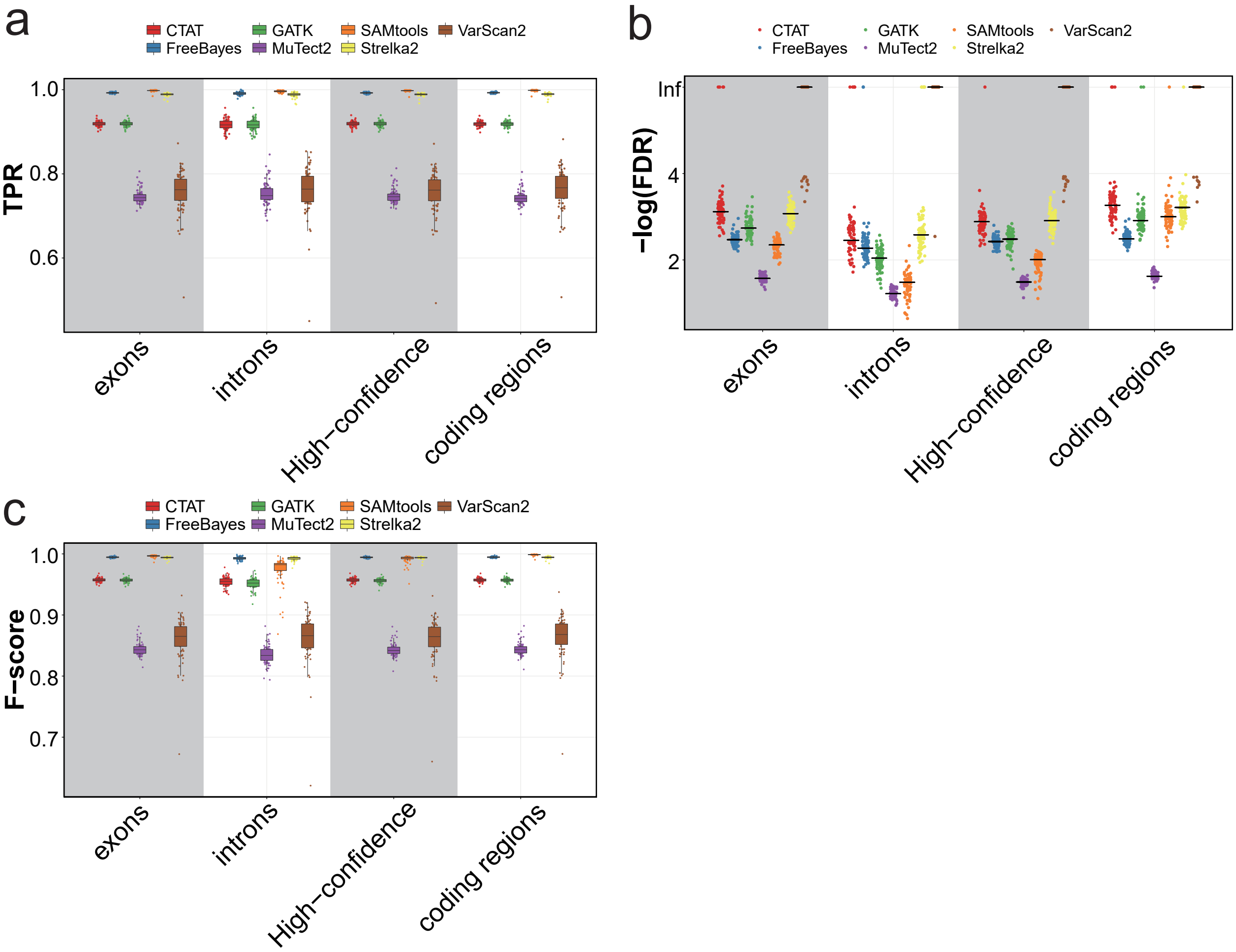

Supplementary Figure 9. The performance measurements of variant-calling tools in different functional genomic regions for simulations based on the SMART-seq2 hepatocellular carcinoma dataset. (a) Boxplots showing the TPRs with minimal read depths ≥ 3 at inserted SNV loci in high-confidence regions. (b) Scatter plot showing the -log transformed FDRs with minimal read depths ≥ 3 at inserted SNV loci in high-confidence regions. The black lines represent the log-transformed median values of FDRs. (c) Boxplots showing the F scores with minimal read depths ≥ 3 at inserted SNV loci in high-confidence regions.

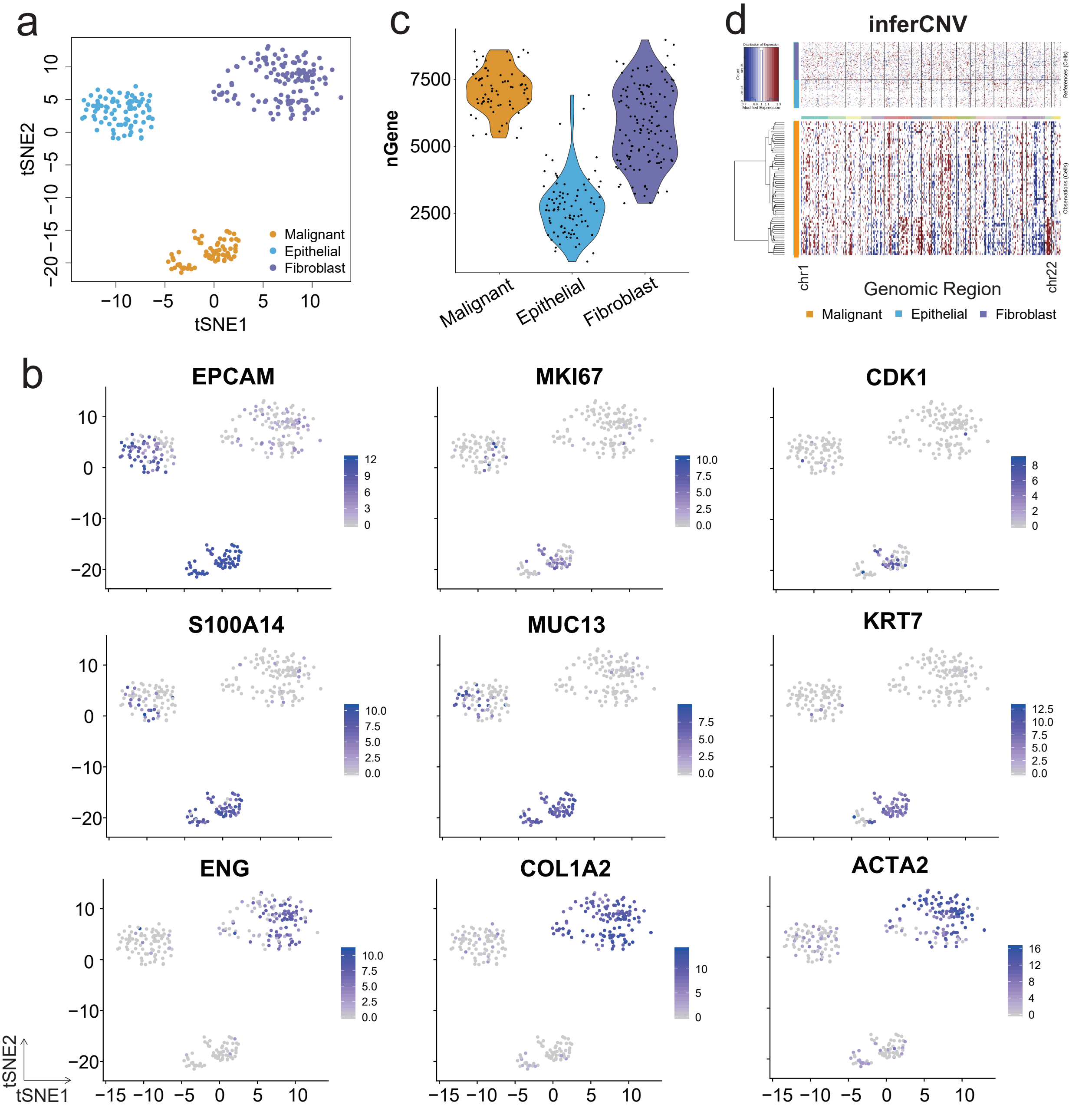

Supplementary Figure 10. Characteristics of CD45⁻ cell subpopulations. (a) t-SNE plots showing the clusters of malignant cells, normal epithelial cells and fibroblasts. (b) t-SNE plots showing expression patterns of selected marker genes. (c) Violin plots showing the number of expressed genes of each cell cluster. (d) Heatmap showing large-scale copy number variations (CNVs) inferred based on transcriptome data of individual cell (row). CNVs in red indicates amplifications, and blue indicates deletions.
