# Supplementary material for: Systematic comparative analysis of single-nucleotide variant detection methods from single-cell RNA sequencing data
Source: Genome Biol. 2019 Nov 19;20:242. doi: 10.1186/s13059-019-1863-4 (PMC6862814; doi:10.1186/s13059-019-1863-4)
Supplement: Supplementary file 3 — Additional file 3. The detailed description of settings for variant calling methods. [file 13059_2019_1863_MOESM3_ESM.docx]

The detailed description of settings for variant calling methods

Here we described the detailed procedures and parameters for the different variant calling methods.

**The GATK Best Practices Pipeline (4.1.0.0):** We followed the best practice guidelines of GATK, as obtained from <http://gatkforums.broadinstitute.org/wdl/discussion/3891/calling-variants-in-rnaseq>. After mapping reads to the reference using GSNAP, we preprocessed the BAM file, including marking duplicates with Picard, splitting, indel realignment, and base recalibration. SNVs were called using HaplotypeCaller (--recover-dangling-heads TRUE, --dont-use-soft-clipped-bases, -stand-call-conf 20.0), and then filtered using VariantFiltration with the recommended parameters (including QD <2.0, FS > 30.0, and a cluster filter).

The LOD threshold (θ_T_) is the key parameter for the GATK pipeline. Here we adjusted the ‘-stand-call-conf’ to 0, 10.0 and 30.0 for parameter adjustment.

The GATK Best Practices Pipeline applied in our benchmarking analysis was currently routinely utilized as SNV calling tool in single cell RNA-seq data, exampled with the Poirion’s method for identification of eeSNVs (effective and expressed nucleotide variations) [13]. Poirion *et al*. has provided an accessible and user-friendly software including the GATK Best Practices Pipeline to call SNVs for single cell RNA-seq data. Notably, we utilized different version of GATK compared to Poirion’s method, although with the same procedures and parameters. Poirion *et al*. used GATK v3.5 in their framework, while we applied GATK v4 in our benchmarking analysis because v4 is now the latest version recommended by the GATK developing group and has been declared to be more reliable than old versions.

**MuTect2 (4.0.4.0):** We preprocessed the BAM file as in the GATK pipeline. However, instead of HaplotypeCaller and VariantFiltration, we performed Mutect2 in tumor-only mode. Although MuTect2 was recommended by its developers to be applied with match normal samples, we still include it in our benchmarking analysis considering its wide usages.

As described by Cibulskis *et al*. (Cibulskis, *et al*. Nature biotechnology 31.3 (2013): 213), the LOD thresholds (θ_T_) are the key parameters for MuTect2. The default value for ‘--tumor-lod-to-emit’ is 3.0 and that for ‘--initial-tumor-lod’ is 2.0. In the parameter adjustment part, we used the parameters of “--tumor-lod-to-emit 0 --initial-tumor-lod 0” and “--tumor-lod-to-emit 5.0 --initial-tumor-lod 5.0”.

**FreeBayes:** Following the guide in <https://github.com/ekg/freebayes>, we run Freebayes with default parameters after sorting and marking duplicates by GATK, then applied the "QUAL > 20" filter as recommended in tutorial.

FreeBayes provides the useful options of ‘-C’ (minimal observations in a single sample, default value = 2) and ‘-F’ (minimal proportion of alternative reads, default value = 0.2) to filter the variants. When we used the parameters of “-C 0 –F 0”, the number of called SNVs became unreasonably large (52110 SNVs for Chr1 in average).

**SAMtools/BCFtools (bcftools-1.9):** We performed variant-calling using mpileup (bcftools version 1.9, options -Q 30 -A -x -Ou) and call (bcftools version 1.9, options -mv).

BCFtools call command provides the useful options of ‘-c’ (minimum allele count), ‘-q’ (minimum allele frequency) to filter the variants.

**Strelka2 (2.9.10.centos6_x86_64):** We performed Strelka2 with default parameters in --rna mode.

No filtering parameters or threshold parameters were provided for Strelka2.

**CTAT:** CTAT was performed using default parameters with --skip_cravat. The vcf file with RNA editing filtered (variants_initial_filtering_clean_snp_RNAedit.vcf.gz) was used for comparison.

No filtering parameters or threshold parameters were provided for CTAT.

**VarScan 2 (v2.4.3):** The mpileup2snp command was applied using default parameters.

VarScan 2 provided the useful parameters of ‘min-coverage’ for minimal read depth (default value = 8), ‘--min-reads2’ for minimal supporting reads (default value = 2), ‘--p-value’ for p-value threshold (default value = 0.99), and ‘--strand-filter’ for whether to ignore variants with >90% support on one strand. When adjusting parameters, we used the settings of ‘--min-coverage 1 --min-reads2 1’ (adjust1), ‘--min-coverage 1 --min-reads2 1 --strand-filter 0’ (adjust2), ‘--min-coverage 1 --min-reads2 1 --strand-filter 0 --p-value 0.95’ (adjust3).
