# Supplementary material for: Systematic comparative analysis of single-nucleotide variant detection methods from single-cell RNA sequencing data
Source: Genome Biol. 2019 Nov 19;20:242. doi: 10.1186/s13059-019-1863-4 (PMC6862814; doi:10.1186/s13059-019-1863-4)
Supplement: Supplementary file 4 — Additional file 4. Review history. [file 13059_2019_1863_MOESM4_ESM.docx]

Review History

**First round of review**

**Reviewer 1**

**Are you able to assess all statistics in the manuscript, including the appropriateness of statistical tests used?**

Yes, and I have assessed the statistics in my report.

**Comments to author:**

Liu et al conducted a systematic comparative analysis of single-nucleotide variants detection methods for analyzing single cell RNA-seq data on both real and simulated data. Broad utilizations of scRNA-seq has granted unprecedented resolution into complicated biological processes and tissues like tumor microenvironment. Deriving identifiable somatic mutations from scRNA-seq is certainly an alternative method in information mining from scRNA-seq data in complementary to the classic transcriptomic profile based cell clustering analysis.
The manuscript is well constructed. The topic is interesting. The authors sequenced the transcriptome of 70 single cell (with using a smart-seq2 protocol) and the WES of matched bulk tumor and healthy tissue from two colorectal cancer patients. Simulation based method evaluation was conducted by RSEM, which in general maintained the complexity of real RNA-seq data. The method evaluation was conducted from multiple aspects, including different mapping platform, different read depth, different datasets and with positive and negative controls. However, from the reviewer's point of view, there are several major concerns that need to be addressed and are listed as follows:
1)      As the author mentioned, calling SNV from scRNA-seq data is certainly with some limitations, like low sequence coverage and complicated alternative splicing events. All the tools in the analysis were in general developed from bulk tissue RNA-seq. Their suitableness for single cell data analysis is questionable. There is at least one tool developed for scRNA-seq SNV calling such as the eeSNVs (Poirion O, et al. Nature communications. 2018 Nov 20;9(1):4892.). Although the SNV calling is conducted by GATK in eeSNVs, their model based filtering strategy is worth to be considered in the comparison.
2)     The SNVs identified from bulk WES data were served as benchmarks or "true mutations" in the comparative analysis. The rationality of using SNV detected from bulk should be evaluated first. 
a.     The authors are expected to show how the two specimens of the single cell and bulk data were collected. Are they from a close region of the tumor?
b.     The authors are also expected to characterize the . On pg7 line 165, the authors mentioned "we only retained homozygous SNPs for benchmarking". Do this mean only the germline homozygous SNPs identified in the normal data are used for comparison? Then why the analysis were conducted on cancer cell? If the benchmark SNPs identified in the bulk data are cancer specific, I suspect how many homozygous SNPs can be identified. If the SNPs identified in the bulk data are just somatic SNVs, considering the tumor may have certain heterogeneity, one mutation may not be seen in all the cells. In summary, the author should offer detailed analysis procedure including how the benchmark mutations are identified and validated, how the statistics were computed from the variants identified in different cells, and if possible, provide the analysis codes.
c.     If the samples and experimental condition permits, experimental validations of the benchmark SNVs are expected.
d.     The author should also make sure if the utilized method is VarScan or VarScan2 since they mentioned on pg19, line 448 "we aligned reads using the BWA-PICARD pipeline and called SNPs using VarScan" while the first version of VarScan is rarely used now. 
3)     The 70 cells were saturately sequenced. However, the SNVs identified from the cell can be still varied, due to (1) un-observed transcripts, and (2) intra-tumoral heterogeneity. The authors are expected to offer a comprehensive evaluation of the variation of SNVs identified in different cells and how the method evaluation statistics were computed by using the varied SNV profiles in different cells. 
4)     Another concern is about how the parameters affect the performance. As mentioned by the authors on pg 17, line 384 "To obtain a relatively fair comparison, we used default parameters of all analyzed tools to call variants." From the reviewer's point of view, the default setting of the methods designed for bulk data analysis were definitely optimized for scRNA-seq data. I understand it is not applicable to derive the optimized parameters for all methods in one work. However, to make a fair comparison, the authors are expected to test how the performance of the methods varied based on their key parameters, such as if the less sensitive MuTect2 can be more sensitive with adjusted parameters.
5)     What is the averaged read depth of ERCC. If it is much higher than real reads, the FDR may be under estimated.
6)     On pg 12, by my understanding, the intronic reads are with a much less abundance than the reads mapped to coding region. If it is true, the true-positive rates will be definitely much lower for SNVs called in introns than the reads of exons. If the read depth was not normalized in the analysis, this comparison does not offer a lot of meaningful information. In addition, since only the WES was measured from real bulk data, how the simulation can generate intronic reads should be discussed. Otherwise, I suggest to remove this part.
7)     Why the detection of heterozygous variants was not compared on the real data? The current experimental design could enable this analysis. 
8)     The 10x data only measure short sequence on the 3' side and the total sequence amount per cell is much smaller than smart-seq2, so definitely the number of detected SNVs were dramatically fewer for 10X. The 10x protocol is not suitable for genomic variants calling due to majority of the coding region won't be sequenced. From the reviewer's personal point of view, it is improper to say "10X could be used to call variants, with high specificities in high- confidence coding regions, although the overall sensitivities were low". 
9)     The manuscript more focused on SNV comparison but somehow ignored the advantage of single cell experiment - large number of cells share certain similarity. An advanced question is what tools we should apply to identify mutations from different cancer cells for a further characterization of the evolutionary trajectory of the cancer cells, like in Poirion et al's work, an application of called SNVs in single cell in identifying subpopulations. I expect the author to discuss more in this point. Such as, how the distributions of the SNVs identified in each single cell vary by different tools. Are some tumor cells with the majorities of true positive SNVs and others do not, or every tumor cell has more or less the same number of true positive SNVs. Does the called SNV for each cell have more frequency in certain genes, like tumor suppressor genes? This can help to authors to better define their results. For the two different questions of (1) identify somatic mutations commonly shared by certain cancer cells versus normal cells, and (2) identify germline mutations shared by all cells, there should be different recommendations of methods.
There are some additional minor comments, mainly regarding the writing:
1)     Are all the sequenced CD45 negative cells cancer cell, or there are stromal cells? The authors are expected to provide a more systematic characterization of the cells used for the method evaluation. 
2)     The y axis of figure 2f should be "true positive rates" rather than "False discovery rates".
3)     The language of manuscript can be improved. More importantly, the analysis procedures of some comparisons were not clearly described. I recommend the authors to move the long "Variant/mutation-calling programs" part into a supplementary method and detail the procedures of the comparisons and analysis in the method part.

**Reviewer 2**

**Are you able to assess all statistics in the manuscript, including the appropriateness of statistical tests used?**

Yes, and I have assessed the statistics in my report.

**Comments to author:**

Please consider the following questions:
- Are the methods appropriate to the aims of the study, are they well described, and are necessary controls included? If not, please specify what is required.

As a benchmark study of various tools for SNV detection from single cell RNA-seq data, the study was very well designed. The study evaluated seven methods including SAMtools, the GATK pipeline, CTAT, FreeBayes, MuTect2, Strelka2 and VarScan, using both simulation and scRNA-seq datasets. The authors considered different alignment tools, different regions of the genome, sequencing depths, etc. The study was very extensive and the conclusions from the study were highly solid. Finally they give valuable suggestions on the SNV detection tools that are most suitable under different conditions. I find the benchmark study timely and useful. 


- Are the conclusions adequately supported by the data shown? If not, please explain

Yes. The study was very extensive and the conclusions are solidly supported by the data. 

- Are sufficient details provided to allow replication and comparison with related analyses that may have been performed? If not, please specify what is required.


The paper was well written and can be relatively duplicated by an investigator with necessary background in genomics. T

- Does the work represent a significant advance over previously published studies?

It is hard to say that the study represents a significant advance because it is a benchmark study. The authors did not invent new methods. Instead, they evaluated other commonly used methods for bulk RNA-seq analysis to single cell RNA-seq data analysis, which has not been done before. Although no new methods were developed, the results from the benchmark study are highly useful for investigators interested in finding SNVs in single cell RNA-seq data. SNVs in single cells are highly important for many studies. 

- Is the paper of broad interest to others in the field, or of outstanding interest to a broad audience of biologists?

The paper is of interests to people working in detecting SNVs in single cell RNA-seq data. 

Following are some detailed comments. 

Although the paper was well written, there are a few typos in the manuscript. Therefore, I would suggest the authors to carefully check the paper to correct such errors. Following are just some I found. 

line 40: remove "when"
line 94: Remove "s" after "SNV"
line 189: remove "y" after "short-read"
lines 189-191: change "aligning" to "alignment" unless this is common use in the field
line 196: remove "when"
line 197: add "for STAR and GSNAP" after TPRs. I would suggest to change "true positive rates" to "TPRs" throughout except the first occurrence. The authors keep changing back and forth for the two usages. 
line 208: I believe "true" should be "false". Please double check. 
line 505: Please define "F-score"
line 662 Figure 1: change the order of subfigure b) and c).

**Authors Response**

**Point-by-point responses to the reviewers’ comments:**

Comment

*Response*

Reviewer #1: 
Liu et al conducted a systematic comparative analysis of single-nucleotide variants detection methods for analyzing single cell RNA-seq data on both real and simulated data. Broad utilizations of scRNA-seq has granted unprecedented resolution into complicated biological processes and tissues like tumor microenvironment. Deriving identifiable somatic mutations from scRNA-seq is certainly an alternative method in information mining from scRNA-seq data in complementary to the classic transcriptomic profile based cell clustering analysis. 
The manuscript is well constructed. The topic is interesting. The authors sequenced the transcriptome of 70 single cell (with using a smart-seq2 protocol) and the WES of matched bulk tumor and healthy tissue from two colorectal cancer patients. Simulation based method evaluation was conducted by RSEM, which in general maintained the complexity of real RNA-seq data. The method evaluation was conducted from multiple aspects, including different mapping platform, different read depth, different datasets and with positive and negative controls. However, from the reviewer's point of view, there are several major concerns that need to be addressed and are listed as follows: 
1) As the author mentioned, calling SNV from scRNA-seq data is certainly with some limitations, like low sequence coverage and complicated alternative splicing events. All the tools in the analysis were in general developed from bulk tissue RNA-seq. Their suitableness for single cell data analysis is questionable. There is at least one tool developed for scRNA-seq SNV calling such as the eeSNVs (Poirion O, et al. Nature communications. 2018 Nov 20;9(1):4892.). Although the SNV calling is conducted by GATK in eeSNVs, their model based filtering strategy is worth to be considered in the comparison. 

*We thank the reviewer for pointing out the importance of our topic and the comprehensive aspects of our method evaluation that will be of general interest to the field of variants calling in single-cell-sequencing data. We also greatly appreciate all the constructive suggestions and criticisms, which have helped us to further improve the quality of our manuscript. 

Although we agreed that the tool developed by Poirion et al. was important to analyze SNVs in scRNA-seq data, their model-based filtering strategy was originally designed to obtain SNVs significantly associated with gene expression, which they defined as eeSNVs (effective and expressed nucleotide variations). We did not try to evaluate whether the SNVs were associated with expression level, since such analyses are out of scope of the current manuscript. As for the SNV calling part of Poirion’s method, the calling method and filtering strategy was exactly the same as the GATK method applied in our benchmarking analysis, except for the subtle difference in software version. Poirion’s method was based on GATK v3.5 while our analysis was based on GATK v4.1.0.0. GATK v4.1, the latest version recommended by the GATK developing group (https://github.com/broadinstitute/gatk/releases), has been declared to be more reliable than old versions, and thus we used the GATK v4.1.0.0 in our work. 

To clarify this point, we have modified our descriptions in Supplementary Methods section (Additional File 3) as “The GATK Best Practices Pipeline applied in our benchmarking analysis was currently routinely utilized as SNV calling tool in single cell RNA-seq data, exampled with the Poirion’s method for identification of eeSNVs (effective and expressed nucleotide variations). Poirion et al. has provided an accessible and user-friendly software including the GATK Best Practices Pipeline to call SNVs for single cell RNA-seq data. Notably, we utilized different version of GATK compared to Poirion’s method, although with the same procedures and parameters. Poirion et al. used GATK v3.5 in their framework, while we applied GATK v4 in our benchmarking analysis because v4 is now the latest version recommended by the GATK developing group and has been declared to be more reliable than old versions.” We also added “SSrGE, developed to link effective and expressed nucleotide variations associated with gene expression based on single cell RNA-seq data, contains a module of identifying variants based on GATK” in the main text.* 


2) The SNVs identified from bulk WES data were served as benchmarks or "true mutations" in the comparative analysis. The rationality of using SNV detected from bulk should be evaluated first. 
a. The authors are expected to show how the two specimens of the single cell and bulk data were collected. Are they from a close region of the tumor? 

*We thank the reviewer for suggesting us to provide more details about sample collection process in our manuscript, which is helpful for clarifying the reliability and consistency of our data. For the two treatment-naïve patients in our study, they were enrolled and pathologically diagnosed with colorectal adenocarcinoma at Peking University People’s Hospital. Fresh tumors and adjacent normal tissue samples were surgically resected from these two patients. Each tissue was cut into two pieces, with one for further single-cell collection and the other for bulk sequencing. This procedure could maximally ensure that the single-cell and bulk sequencing data were generated from a close region of the tumor/normal tissue. The details of such experimental procedures have been added in the revised Methods as “Briefly, fresh tumors were surgically resected from two treatment-naïve patients pathologically diagnosed with colorectal adenocarcinoma. Each tissue was cut into two pieces, with one for further single-cell collection and the other for bulk sequencing. This procedure could maximally ensure that the single-cell and bulk sequencing data were generated from a close region of the tissue.”* 


b. The authors are also expected to characterize the . On pg7 line 165, the authors mentioned "we only retained homozygous SNPs for benchmarking". Do this mean only the germline homozygous SNPs identified in the normal data are used for comparison? Then why the analysis were conducted on cancer cell? If the benchmark SNPs identified in the bulk data are cancer specific, I suspect how many homozygous SNPs can be identified. If the SNPs identified in the bulk data are just somatic SNVs, considering the tumor may have certain heterogeneity, one mutation may not be seen in all the cells. In summary, the author should offer detailed analysis procedure including how the benchmark mutations are identified and validated, how the statistics were computed from the variants identified in different cells, and if possible, provide the analysis codes. 

*We appreciate such important comments. As suggested by the reviewer, we have now provided more detailed analysis procedures and performed additional studies to further clarify the identification of germline SNPs or somatic SNVs. 

It should be noticed that our analyses contained two parts. The first part, “performance evaluation of variant callers on real data” as in the main text, was designed to evaluate the performances of different variant calling tools for identification of germline SNPs. Because of the heterogeneity of tumors as the reviewer mentioned, single tumor cell would harbor different variants, and thus it is not feasible to perform performance evaluation based on overall true variants (including germline SNPs and somatic SNVs) on real data. However, performance evaluation based on germline SNPs is feasible since germline SNPs identified with bulk Exome-seq are expected to exist in every cancer cell. Thus, we evaluated the performances of different SNV calling tools for identification of germline SNPs and calculated TPRs for each cancer cell as the proportion of identified SNPs using scRNA-seq versus those identified using bulk Exome-seq. 

The other part of our analysis was based on simulation. In this case, we randomly inserted different variants, representing somatic SNVs, in each tumor cells, and the original variants (germline SNPs or SNVs) for each cell were flagged in order to calculate TPRs or FDRs based on the known inserted variants. TPRs were calculated as the proportion of identified random variants in all the inserted variants for each cell, while FDRs were calculated as the proportion of wrong variants in all called variants. 

Notably, the TPRs calculated based on simulations were highly correlated with those calculated based on germline SNPs in real data (Pearson correlation coefficient = 0.958), suggesting the performance consistency of SNVs calling tools for identification of germline SNPs and somatic SNVs in scRNA-seq data. 

We have now added the corresponding clarification in the main text.* 


c. If the samples and experimental condition permits, experimental validations of the benchmark SNVs are expected. 

*We appreciate this important point the reviewer has raised. Following this suggestion, we have validated the reliability of our benchmark SNVs by detecting SNVs from bulk RNA-seq data. We found that of the 16,705 SNVs called from bulk exome-seq data, which were used as golden standard in our analysis, 5,861 were sufficiently expressed (read depths > 5) in RNA-seq data, and 97.8% (5,827/5,861) of them were independently identified from bulk RNA-seq data, supporting the reliability of our benchmark SNVs. We have added the descriptions of such verification in our revised manuscript.* 


d. The author should also make sure if the utilized method is VarScan or VarScan2 since they mentioned on pg19, line 448 "we aligned reads using the BWA-PICARD pipeline and called SNPs using VarScan" while the first version of VarScan is rarely used now. 

*We thank the reviewer for pointing out this oversight. Indeed, we used VarScan2 (v2.4.3) in our analysis, and have now clarified it in the revised manuscript.* 


3) The 70 cells were saturately sequenced. However, the SNVs identified from the cell can be still varied, due to (1) un-observed transcripts, and (2) intra-tumoral heterogeneity. The authors are expected to offer a comprehensive evaluation of the variation of SNVs identified in different cells and how the method evaluation statistics were computed by using the varied SNV profiles in different cells. 

*We greatly appreciate the constructive comments and suggestions, and apologize that we did not adequately describe our methodology. To address these concerns, we have performed further analysis, and provided comparative information about SNVs identified in different cells. We observed variance of the number of identified SNVs in different cells (Additional file 2: Figure S6a). In addition, we compared the number of SNVs shared between cells in each patient and observed substantial sharing of SNVs across multiple cells, as well as non-overlapping distribution of SNVs across cells (Additional file 2: Figure S6b, c), suggesting un-observed transcripts or intra-tumoral heterogeneity, as the reviewer mentioned. 

As for the method evaluation statistics, which were also described in response to 2b), we used germline SNPs identified by bulk Exome-seq (in real data part) or inserted SNVs (in simulation part) as benchmark. Although the SNV profiles are varied, germline SNPs are stable among single cells, and the inserted SNVs are known though they are different among cells in the simulation part.* 


4) Another concern is about how the parameters affect the performance. As mentioned by the authors on pg 17, line 384 "To obtain a relatively fair comparison, we used default parameters of all analyzed tools to call variants." From the reviewer's point of view, the default setting of the methods designed for bulk data analysis were definitely optimized for scRNA-seq data. I understand it is not applicable to derive the optimized parameters for all methods in one work. However, to make a fair comparison, the authors are expected to test how the performance of the methods varied based on their key parameters, such as if the less sensitive MuTect2 can be more sensitive with adjusted parameters. 
 *We appreciate the insightful comments by the reviewer. Following the helpful suggestions, we have investigated the impact of key parameters of different SNV-detecting tools in Additional file 3, and have added corresponding descriptions in our revised manuscript. 

As described by Cibulskis et al. (Cibulskis, et al. Nature biotechnology 31.3 (2013): 213), the log-odds (LOD) thresholds (θT) is the key parameter for MuTect2. We tuned this parameter and found that both sensitivities and FDRs would decrease with higher LOD thresholds. In addition, when the thresholds was reduced to 0, the performance became worse than those with default settings (Additional file 2: Figure S2a, b). 

For the GATK Best Practices Pipeline, the FDRs would change according to the LOD thresholds, while the sensitivities would not be influenced as much (Additional file 2: Figure S2c, d). We have also adjusted the parameters of VarScan2, and found that both the sensitivities and FDRs would increase with the adjusted parameters (Additional file 2: Figure S2e, f). 

In brief, adjusting parameters were important for SNV-calling tools to achieve best performance, and users should choose the most suitable parameters according to the preference of sensitivities or specificities.* 


5) What is the averaged read depth of ERCC. If it is much higher than real reads, the FDR may be under estimated. 
 *Following the helpful suggestions by the reviewer, we have performed comparative analysis, and found that the average read depths of ERCC are much higher than those of real reads (Additional file 2: Figure S1c), as mentioned by the reviewer. Nonetheless, we did not observe any association between read depth and FDRs according to our simulation analysis. To address this concern, we have added a reminder in the main text as “Importantly, the averaged read depths of ERCC are much higher than those of RNA reads, which could lead to potential biases when extending the ERCC-based conclusions to real reads.”* 


6) On pg 12, by my understanding, the intronic reads are with a much less abundance than the reads mapped to coding region. If it is true, the true-positive rates will be definitely much lower for SNVs called in introns than the reads of exons. If the read depth was not normalized in the analysis, this comparison does not offer a lot of meaningful information. In addition, since only the WES was measured from real bulk data, how the simulation can generate intronic reads should be discussed. Otherwise, I suggest to remove this part. 
 *This is an excellent point and we thank the reviewer for providing specific suggestion about the read depth. In fact, one of the important conclusions of our study is that read depth would largely impact the SNV-calling performance. Following the helpful suggestion by the reviewer, we have now performed further analysis and made several revisions in our manuscript. As shown in Fig. 4a, the sensitivities are similar for calling variants from introns and those from exons when controlling minimal read depths. 

The reviewer is correct in pointing out that only the WES was measured from real bulk data. Indeed, the intron part was based on simulation by inserting random variants and WES data was not used here. 

We thank the reviewer for helping us improve our manuscript.* 


7) Why the detection of heterozygous variants was not compared on the real data? The current experimental design could enable this analysis. 

*Regarding the heterozygous variants, we did not analyze them in our original version because the actual expressed bases for these variants are unknown in RNA level. Specifically, when a heterozygous variant could be identified in WES but not in RNA-seq, it is not clear whether this difference is because of the lack of sensitivities for the variant-calling method, or because of allele-specific expression of the related gene. However, heterozygous variants do demonstrate clinical significance in diseases, and thus we have further strengthened our study as the reviewer suggested and have analyzed all the SNPs including both heterozygous and homozygous in this revision. We found that the overall TPRs decreased as expected due to probably fewer mutant reads (Additional file 2: Figure S1b). Notably, the relative orders of TPRs for different tools were similar as those based on homozygous SNPs (Additional file 2: Figure S1b). 

To make this point clearer, we have now extended the descriptions in our main text as: “Genetic variants can be classified into homozygous and heterozygous variants, both of which could provide valuable insights on gene function and could cause pathogenic phenotypes. However, the heterozygous variants might be inconsistent between Exome-seq and RNA-seq data, due to either the lack of sensitivities of the variant-calling methods or the widespread allele-specific expression. Therefore, we mainly focused on homozygous SNPs for benchmarking, unless explicitly stated in certain parts.”* 


8) The 10x data only measure short sequence on the 3' side and the total sequence amount per cell is much smaller than smart-seq2, so definitely the number of detected SNVs were dramatically fewer for 10X. The 10x protocol is not suitable for genomic variants calling due to majority of the coding region won't be sequenced. From the reviewer's personal point of view, it is improper to say "10X could be used to call variants, with high specificities in high-confidence coding regions, although the overall sensitivities were low". 
 *We agreed with the reviewer that the majority of the coding region could not be sequenced for 10X, which limits the applications of SNV identification. Although detecting SNVs with 10X data cannot profile the landscape of all variants, part of variants could still work in several applications like lineage tracing. 

Following the reviewer’s suggestion, in the revised manuscript, we have clarified this point by adding: “Our results showed that the overall sensitivities of SNVs detection in 10X scRNA-seq data were relatively low, potentially due to the low coverages in vast regions of genome. Notably, the sensitivities increase with high specificities when the supporting read depths increase in high-confidence coding regions.” and “Although identifying SNVs using 10X data could not profile the global landscape of variants, it could still be useful in certain conditions such as lineage tracing.”* 


9) The manuscript more focused on SNV comparison but somehow ignored the advantage of single cell experiment - large number of cells share certain similarity. An advanced question is what tools we should apply to identify mutations from different cancer cells for a further characterization of the evolutionary trajectory of the cancer cells, like in Poirion et al's work, an application of called SNVs in single cell in identifying subpopulations. I expect the author to discuss more in this point. Such as, how the distributions of the SNVs identified in each single cell vary by different tools. Are some tumor cells with the majorities of true positive SNVs and others do not, or every tumor cell has more or less the same number of true positive SNVs. Does the called SNV for each cell have more frequency in certain genes, like tumor suppressor genes? This can help to authors to better define their results. For the two different questions of (1) identify somatic mutations commonly shared by certain cancer cells versus normal cells, and (2) identify germline mutations shared by all cells, there should be different recommendations of methods. 

*We appreciate such constructive comments. 

Regarding the SNV-based subpopulation identification, we used the Barnes-Hut t-Distributed Stochastic Neighbor Embedding (t-SNE) for dimensionality reduction and performed K-means clustering on SNVs profiles. Indeed, we found that the 70 cells were clustered into two subpopulations, coordinating to the patient origins. In addition, most tools except for SAMtools achieved better performances than analyses based on gene expression (Additional file 2: Figure S7). Therefore, SNVs could serve as important source for subpopulation identification, consistent with Poirion’s report (Poirion, et al. Nature communications 9.1 (2018): 4892). Specifically, VarScan2 achieved the best clustering result (Average silhouette width: 0.76) while SAMtools showed the worst (Average silhouette width: 0.38). Notably, the clustering methods could be complex and more detailed investigation was needed for further in-depth characterization. 

We further characterized the distribution of true positive SNVs in single cells by comparing the SNVs identified in scRNA-seq with those identified in bulk Exome-seq, representing true positive set. Importantly, the single tumor cells could be clustered by patient origin based on the true positive SNV profiles, and for the same patient, every tumor cell has more or less the same number of true positive SNVs. These observations are consistent with the result of SNV-based subpopulation identification, because we only identified patient-derived subpopulations but did not detect further subpopulations within each patient. 

To answer the question about SNV-enriched gene categories such as the tumor suppressor genes, we compared the frequencies of SNVs enriched in tumor suppressor genes (downloaded from the TSGene2.0) with the baseline frequency of tumor suppressor genes in all genes. To exclude the potential biases caused by enrichment of suppressors in coding genes, we only focused on protein-coding genes in this analysis. We did observe that SNVs identified in single tumor cells were enriched in tumor suppressor genes, which might hint clues for the mechanisms of tumorigenesis. 

Accordingly, we also characterized the proportions of COSMIC mutations in our single cell SNVs. We found that the proportions of COSMIC mutations in malignant cells were much higher than those in non-malignant cells for all tested tools except for MuTect2 (Additional file 2: Figure S6f). 

It is reasonable to concern whether somatic mutations and germline mutations would have different recommendations of methods, as mentioned by the reviewer. Indeed, we performed one-sample analysis to identify SNVs in our study, and theoretically, one-sample analysis cannot discriminate whether a mutation is shared by all cells or part of cells. Although germline SNPs were used in bulk data-based analysis, while simulated somatic SNVs were used in simulation based analysis, similar results were obtained and the orders of sensitivity for different SNV calling tools were highly correlated (Pearson correlation coefficient = 0.958). Therefore, we recommended the same tools for identification of germline SNPs and somatic SNVs from one-sample scRNA-seq. We have clarified this point in Discussion. 

We appreciate this important point the reviewer has raised, which helped us to significantly strengthen the comprehensive characterizations of single cell SNVs in our study. We have revised our manuscript to include the new analysis.* 


There are some additional minor comments, mainly regarding the writing: 
1) Are all the sequenced CD45 negative cells cancer cell, or there are stromal cells? The authors are expected to provide a more systematic characterization of the cells used for the method evaluation. 

*We apologize for the inadequate description about the sample chrematistics in our previous version of manuscript. Indeed, we obtained 291 CD45- cells in total from other CRC-related project in our lab, and 70 of them were defined as malignant cells, which were used in this study. We used the malignant cells in our analysis in order to provide similar genomic background as in most applications of SNV detection. 

To reveal the intrinsic structures and potential subtypes, we used t-SNE for dimensionality reduction and performed K-means clustering on all the sequenced 291 CD45- cells. As a result, cells were partitioned into three clusters (Additional file 2: Figure S10a), including fibroblasts, epithelial and malignant cells, each with unique signature genes. Fibroblasts highly expressed classical makers of ENG, COL1A2 and ACTA2 (Additional file 2: Figure S10b), while the remaining two clusters were composed of epithelial cells, characterized by the high expression of the Epithelial Cell Adhesion Molecule (EPCAM). Notably, one of the two clusters was characterized by specific expression of cell cycle related genes including MKI67 and CDK1, as well as cancer-associated genes including S100A14, MUC13 and KRT7, and thus was defined as malignant cells (Additional file 2: Figure S10b). The malignant cell cluster harbored much higher number of expressed genes (Additional file 2: Figure S10c) and showed large-scale chromosomal copy-number variations inferred based on the transcriptome data (Additional file 2: Figure S10d), which further confirming the malignant phenotype of this cluster. In addition, we called SNVs from all sequenced CD45- cells, and found that the number of SNVs in malignant cells were much higher than those in epithelial cells or fibroblasts for all variant-calling methods except for VarScan2 (Additional file 2: Figure S6d). This difference might due to the low sensitivities of VarScan2 in low read depth condition, because a large number of SNVs in malignant cells were with low read depths, which might be related with copy number variations or nonsense-mediated mRNA decay (Additional file 2: Figure S6e). 

We thank the reviewer for bringing up this topic and we believe that our added analyses will enhance the continuity of this study.* 


2) The y axis of figure 2f should be "true positive rates" rather than "False discovery rates". 

*We thank the reviewer for this suggestion, and we have revised it.* 


3) The language of manuscript can be improved. More importantly, the analysis procedures of some comparisons were not clearly described. I recommend the authors to move the long "Variant/mutation-calling programs" part into a supplementary method and detail the procedures of the comparisons and analysis in the method part. 

*Following the reviewer’s suggestions, we have now moved the "Variant/mutation-calling programs" part into a supplementary method and described the procedures of the comparisons and analyses in detail.* 


Reviewer #2: === 
This box is for your report. All comments included here will be passed to the authors. 
=== 
Please consider the following questions: 
- Are the methods appropriate to the aims of the study, are they well described, and are necessary controls included? If not, please specify what is required. 

As a benchmark study of various tools for SNV detection from single cell RNA-seq data, the study was very well designed. The study evaluated seven methods including SAMtools, the GATK pipeline, CTAT, FreeBayes, MuTect2, Strelka2 and VarScan, using both simulation and scRNA-seq datasets. The authors considered different alignment tools, different regions of the genome, sequencing depths, etc. The study was very extensive and the conclusions from the study were highly solid. Finally they give valuable suggestions on the SNV detection tools that are most suitable under different conditions. I find the benchmark study timely and useful. 

- Are the conclusions adequately supported by the data shown? If not, please explain 

Yes. The study was very extensive and the conclusions are solidly supported by the data. 

- Are sufficient details provided to allow replication and comparison with related analyses that may have been performed? If not, please specify what is required. 


The paper was well written and can be relatively duplicated by an investigator with necessary background in genomics. T 

- Does the work represent a significant advance over previously published studies? 

It is hard to say that the study represents a significant advance because it is a benchmark study. The authors did not invent new methods. Instead, they evaluated other commonly used methods for bulk RNA-seq analysis to single cell RNA-seq data analysis, which has not been done before. Although no new methods were developed, the results from the benchmark study are highly useful for investigators interested in finding SNVs in single cell RNA-seq data. SNVs in single cells are highly important for many studies. 

- Is the paper of broad interest to others in the field, or of outstanding interest to a broad audience of biologists? 

The paper is of interests to people working in detecting SNVs in single cell RNA-seq data. 

Following are some detailed comments. 

Although the paper was well written, there are a few typos in the manuscript. Therefore, I would suggest the authors to carefully check the paper to correct such errors. Following are just some I found. 

line 40: remove "when" 

*We thank the reviewer and we have revised it.* 


line 94: Remove "s" after "SNV" 

*We thank the reviewer and we have revised it.* 


line 189: remove "y" after "short-read" 

*We thank the reviewer and we have revised it.* 


lines 189-191: change "aligning" to "alignment" unless this is common use in the field 

*We thank the reviewer and we have revised it.* 


line 196: remove "when" 

*We thank the reviewer and we have revised it.* 


line 197: add "for STAR and GSNAP" after TPRs. I would suggest to change "true positive rates" to "TPRs" throughout except the first occurrence. The authors keep changing back and forth for the two usages. 

*We thank the reviewer for pointing out this inconsistency. We have now changed "true positive rates" to "TPRs" and “false discovery rates” to “FDRs” throughout except the first occurrence.* 


line 208: I believe "true" should be "false". Please double check. 

*We thank the reviewer and have revised it.* 


line 505: Please define "F-score" 

*We thank the reviewer and have added the definition of F-score: 
“F-score = 2 * Specificity * Sensitivity / (Specificity + Sensitivity)”* 


line 662 Figure 1: change the order of subfigure b) and c). 

*We thank the reviewer and have refined the order of figures here.*

**Second round of review**

**Reviewer 1**

The authors addressed reviewers’ comments well by putting a lot of effort. The revised version is improved in quality significantly. I have no further revision to suggest.

I believe this is a very good exploration for analyzing genomic mutation from single-cell sequencing data, where a comprehensive evaluation is really needed. Whether using scRNA-seq data to call mutation will be popular remains to be seen. It would be great if the authors can make a user-friendly recommendation and pipeline, in the future, to enable an easy mutation call from scRNA-seq data, and more importantly, the significance assessment and in silicon validations.
